# The physiological variability of channel density in hippocampal CA1 pyramidal cells and interneurons explored using a unified data-driven modeling workflow

**DOI:** 10.1371/journal.pcbi.1006423

**Published:** 2018-09-17

**Authors:** Rosanna Migliore, Carmen A. Lupascu, Luca L. Bologna, Armando Romani, Jean-Denis Courcol, Stefano Antonel, Werner A. H. Van Geit, Alex M. Thomson, Audrey Mercer, Sigrun Lange, Joanne Falck, Christian A. Rössert, Ying Shi, Olivier Hagens, Maurizio Pezzoli, Tamas F. Freund, Szabolcs Kali, Eilif B. Muller, Felix Schürmann, Henry Markram, Michele Migliore

**Affiliations:** 1 Institute of Biophysics, National Research Council, Palermo, Italy; 2 Blue Brain Project, École Polytechnique Fédérale de Lausanne, Campus Biotech, Geneva, Switzerland; 3 University College London, London, United Kingdom; 4 University of Westminster, London, United Kingdom; 5 Laboratory of Neural Microcircuitry (LNMC), Brain Mind Institute, EPFL, Lausanne, Switzerland; 6 Institute of Experimental Medicine, Hungarian Academy of Sciences, Budapest, Hungary; 7 Faculty of Information Technology and Bionics, Pázmány Péter Catholic University, Budapest, Hungary; SUNY Downstate MC, UNITED STATES

## Abstract

Every neuron is part of a network, exerting its function by transforming multiple spatiotemporal synaptic input patterns into a single spiking output. This function is specified by the particular shape and passive electrical properties of the neuronal membrane, and the composition and spatial distribution of ion channels across its processes. For a variety of physiological or pathological reasons, the intrinsic input/output function may change during a neuron’s lifetime. This process results in high variability in the peak specific conductance of ion channels in individual neurons. The mechanisms responsible for this variability are not well understood, although there are clear indications from experiments and modeling that degeneracy and correlation among multiple channels may be involved. Here, we studied this issue in biophysical models of hippocampal CA1 pyramidal neurons and interneurons. Using a unified data-driven simulation workflow and starting from a set of experimental recordings and morphological reconstructions obtained from rats, we built and analyzed several ensembles of morphologically and biophysically accurate single cell models with intrinsic electrophysiological properties consistent with experimental findings. The results suggest that the set of conductances expressed in any given hippocampal neuron may be considered as belonging to two groups: one subset is responsible for the major characteristics of the firing behavior in each population and the other is responsible for a robust degeneracy. Analysis of the model neurons suggests several experimentally testable predictions related to the combination and relative proportion of the different conductances that should be expressed on the membrane of different types of neurons for them to fulfill their role in the hippocampus circuitry.

## Author summary

The peak conductance of many ion channel types measured in any given animal is highly variable across neurons, both within and between neuronal populations. The current view is that this occurs because a neuron needs to adapt its intrinsic electrophysiological properties either to maintain the same operative range in the presence of abnormal inputs or to compensate for the effects of pathological conditions. Limited experimental and modeling evidence suggests this might be implemented via the correlation and/or degeneracy in the function of multiple types of conductances. To study this mechanism in hippocampal CA1 neurons and interneurons, we systematically generated a set of morphologically and biophysically accurate models. We then analyzed the ensembles of peak conductance obtained for each model neuron. The results suggest that the set of conductances expressed in the various neuron types may be divided into two groups: one group is responsible for the major characteristics of the firing behavior in each population and the other is more involved with degeneracy. These models provide experimentally testable predictions on the combination and relative proportion of the different conductance types that should be present in hippocampal CA1 pyramidal cells and interneurons.

## Introduction

Any given neuron in the brain is part of a network, in which it exerts its action by transforming the input it receives into an output. This function is specified by the particular shape and passive electrical properties of the neuronal membrane, the composition and spatial distribution of ion channels across its processes, and the functional properties of the synaptic inputs themselves. During development and during the entire lifetime of a neuron, its input/output function is adapted to realize ongoing refinement of the function of the neuron and circuit, or maintain functional robustness in the face of constant protein turnover or an evolving pathological condition. Such adaptability of individual neurons can be achieved through a myriad of dynamic mechanisms, including structural, intrinsic, and synaptic plasticity. A direct experimental evidence for these mechanisms is the high variability observed for the current generated by specific types of ion channels measured across individual neurons, from either a homogeneous population or different cell populations (e.g. [1]). The mechanisms responsible for this variability are not well understood, although there are clear experimental and modeling indications that correlation and degeneracy among a variety of conductances can be involved [2,3]. The phenomenon of degeneracy allows the possibility, for a complex biological system, to perform the same function using structurally different elements [4]. In the context considered in this paper, it refers to the robust and tunable adjustment of a neuron’s firing properties [5]. For example, a neuron can be tuned to perform a given function by expressing in the membrane a specific set of conductances with a specific dendritic distribution (Migliore (2003)); degeneracy can result in this tuning being robust, by implementing the same function with many different configurations of the same set of conductances. This property has been systematically studied in crab stomatogastric ganglion neurons [2, 6] and in Globus Pallidus neurons of the rat [7]. In the present study, we investigate this issue for neurons of the hippocampal CA1 region. These neurons are important because they have a critical position as the main output stage of the hippocampal circuitry [8]. The hippocampal CA1 pyramidal neurons, in particular, exhibit a peculiar ensemble and distribution of conductances (reviewed in [9]), subject to significant changes following activity-dependent biochemical processes, such as activation of protein kinase A and C, or Ca/calmodulin dependent kinase II [10, 11, 12], pathological conditions (e.g. [13, 14]), or traumatic brain injuries [15, 16]. There must then be an extremely robust compensatory mechanism in these neurons, or in the network, which maintains or re-establishes the physiological activity within an operation range, in spite of a potentially large change in its intrinsic properties or synaptic input. Here we study the mechanisms of robustness of intrinsic properties by using a unified data-driven workflow and open source analysis and simulation tools. From a set of experimental recordings and morphological reconstructions, we implemented many morphologically and biophysically accurate models for CA1 pyramidal neurons and interneurons, with intrinsic electrophysiological properties constrained by and consistent with the experimental findings. The results indicate that a few currents need to be expressed at a relatively stable level, whereas others can be expressed within a much wider range. The analysis of the model neurons suggests many specific experimentally testable predictions on the combination and relative proportion of the different ionic conductances, and their relationship to robustness of intrinsic properties.

## Results

### Experimental data used for modeling

**Fig 1:**
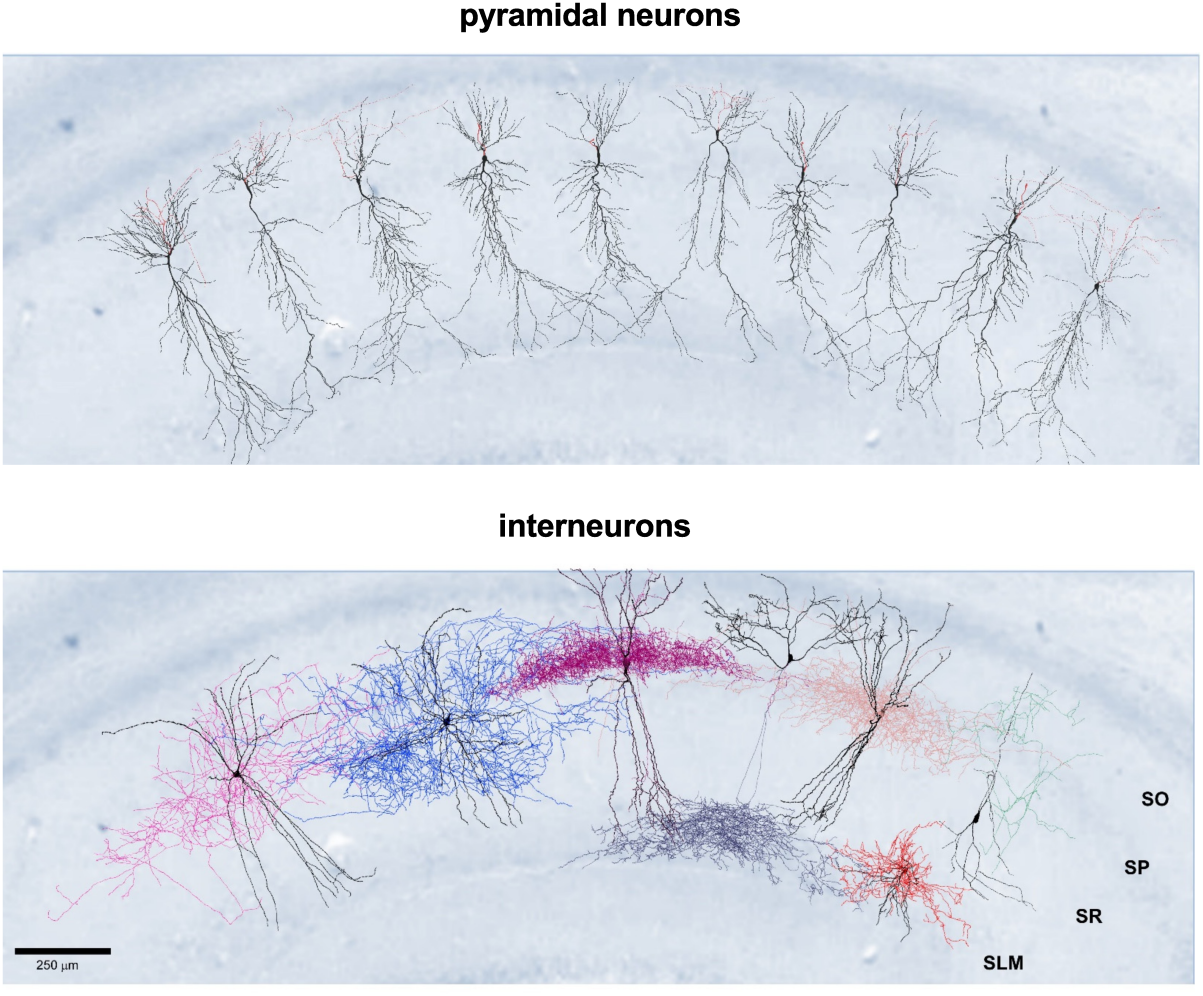
The 3D reconstructions of CA1 cells in rat hippocampus used in this study. (Top) ***Pyramidal cells;*** dendrites are shown in black, axons in red; cell identifier, from left: 990803, oh140807_A0_idJ, oh140807_A0_idH, oh140807_A0_idG, oh140807_A0_idF, 050921AM2, oh140807_A0_idC, oh140807_A0_idB, oh140807_A0_idA; (Bottom) ***Interneurons***, from left to right: basket cell (dendrites in black, axon in pink [Cell number 990111HP2]); bistratified cell (dendrites in black, axon in blue [Cell number 980513B]); axo-axonic cell (dendrites in black, axon in purple [Cell number 970911C]); OLM cell (dendrites in black, axon in dark blue [Cell number 011017HP2]); Ivy cell (dendrites in black; axon in light pink [Cell number 010710HP2]); perforant path associated cell (dendrites in black, axon in red [Cell number 011127HP1]); Schaffer collateral-associated cell (dendrites in black, axon in green [Cell number 990827IN5HP3]). Reconstructions by Joanne Falck and Sigrun Lange. SO Stratum Oriens, SP Stratum Pyramidale, SR Stratum Radiatum, SLM Stratum Lacunosum-Moleculare. 3D reconstructions of the PPA, OLM, axo-axonic cells and of other examples of different types of cells are available in S1 Fig of Mercer and Thomson [17].

To implement a set of data-driven neuron models, we start from a set of morphological reconstructions of neurons and somatic voltage traces obtained from *in vitro* slice preparations of rat hippocampal tissue to use as constraints (see Methods). In Fig 1 we show several examples of the 34 morphologies used in this work (19 pyramidal cells and 15 interneurons), superimposed on a rat hippocampal slice stained for parvalbumin for illustrative purposes.

**Fig 2:**
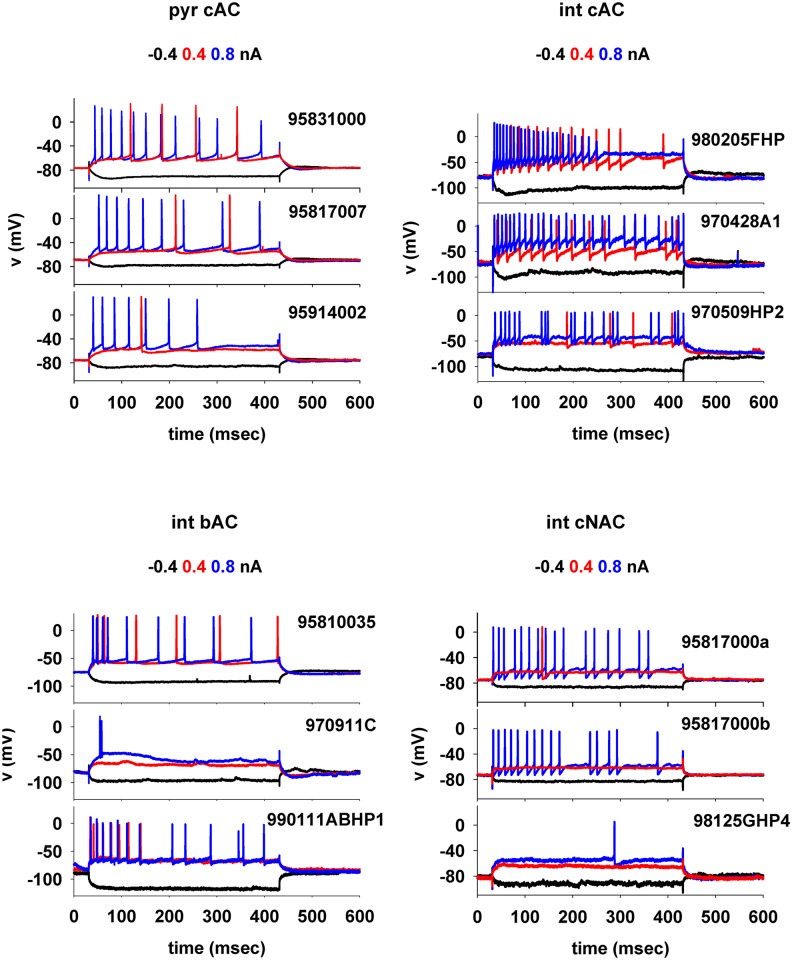
Experimental voltage traces used for the optimization pipeline. (Top) Typical somatic traces obtained during a step current stimulation protocol (-0.4, 0.4 and 0.8 nA for 400 ms) from intracellular recordings performed using sharp electrodes on CA1 pyramidal cells (left) and interneurons (right) classified as continuous accommodating cells (*cAC*); (bottom) typical traces from interneurons classified as bursting accommodating, *bAC*, (left) and continuous non-accommodating, *cNAC*, (right) cells [18].

A total number of 1456 experimentally obtained somatic voltage traces for a range of stimulation protocols were used in the optimization pipeline to constrain the models (see Methods). Collections of traces for individual neurons were manually assigned to four electrical types (e-type), according to the firing pattern exhibited during increasing somatic current injections [18], and using the classification proposed in the Petilla convention [19]. The 832 traces from pyramidal neurons, with an increasing inter-spike-interval (ISI), were all classified as continuous accommodating cells (*cAC)*. For interneurons, 240 traces were classified as *cAC*, 160 traces as bursting accommodating cells (*bAC)*, and 224 traces, whose firing rate is constant, as continuous non-accommodating cells (*cNAC)*. Typical examples illustrating the physiological variability observed for these e-types are shown in Fig 2. A more quantitative analysis and comparison of their features will be presented elsewhere (Bologna et al., manuscript in preparation). Different pyramidal neurons (Fig 2, *pyr cAC*) exhibited significantly different responses to the same input. For example, a near-threshold 0.4 nA somatic current injection may or may not generate a few action potentials, whereas a 0.8nA input can result in a 2-fold range for the number of elicited action potentials (APs) (Fig 2, *pyr cAC*, blue traces). Interneurons classified as *cAC* also exhibited a large inter-cell variability, with different cells responding to the same stimulus with a wide range of spike patterns, such as tonic firing (Fig 2, *int cAC* plots, cell *970428A1*), stuttering (cell *970509HP2*), and depolarization block (cell *980205FHP*). The other two interneuron e-types, *bAC* and *cNAC*, also exhibited a large variability among different cells (Fig 2, *bottom plots*). This variability can be the result of different morphologies and/or a different density and distribution of the conductances expressed on the membrane of the different neurons. In the following sections, we will explore in more detail this issue by implementing and analyzing cellular level models that are able to reproduce these results.

### Model optimization

For each e-type (see S1–S4 Tables and Methods), a set of electrophysiological features were extracted from all voltage traces belonging to that e-type. All the pyramidal cell morphologies were used to implement *cAC* models, whereas interneuron morphologies were used to obtain *cAC*, *cNAC*, and *bAC* models following the known firing behavior of each type of morphology (see legend of Fig 1 and S5 Table). Features and morphologies were then used to obtain a set of optimized models for each e-type, using a heuristic parameter optimization process that employed multi-objective genetic algorithms. Each optimization run (see Methods for details) returned a number of viable “individuals”, each one with a specific ensemble of peak ion channel conductance and passive properties consistent with the chosen “objectives” (i.e. a set of experimental features). As a cost function for the optimization process we used a score defined by the total error associated with each individual, calculated as the sum of the absolute deviations of model features from the experimental mean, in units of the experimental standard deviation (*sd*) obtained for the value of each objective. A score = 0 would correspond to an individual with all parameters equal to the average value of the corresponding experimental electrophysiological feature. The total error thus gave an idea of how good the individual was in representing the neuron’s overall expected behavior under a series of 400 ms long somatic current injection steps. The final choice to accept an individual as a plausible representation of a given e-type was based on the error obtained for each objective. An individual with a *sd*<2 for all objectives was considered acceptable.

**Fig 3:**
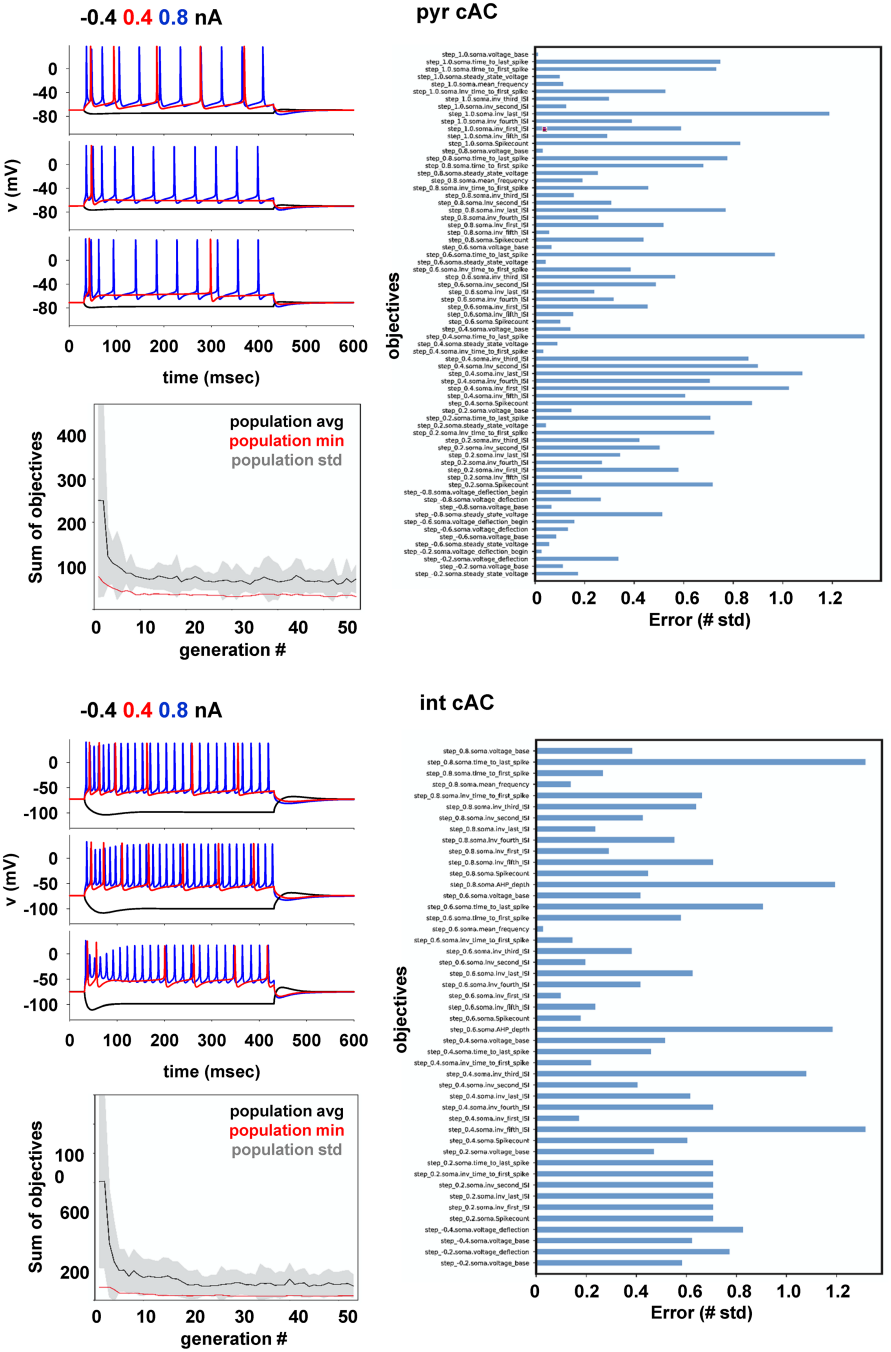
Model optimization. Typical optimization results for *cAC* pyramidal cells (top) and interneurons (bottom). The top left graph of each panel shows a few examples of model traces from three individuals during a current injection of -0.4, 0.4, and 0.8 nA (black, red, and blue traces, respectively). The right graph of each panel reports the objective scores for the best individual. The bottom left graph in each panel shows a typical evolution of the total score during an optimization run.

Typical optimization results for pyramidal and interneuron *cAC* e-types are shown in Fig 3. Traces obtained for different somatic current injections from three individuals (Fig 3, traces on top left graph of each panel), showed that the optimization process was able to take into account the experimental variability. Different individuals exhibited significantly different responses to the same stimulus, as in the experiments. The evolution of the total score as a function of the number of generations in the optimization process (bottom graph in each panel), showed that the optimization converged nearly monotonically in relatively few iterations, having reached a relatively stable minimum within approximately 60 generations. The list of objective scores for the best individual in each case (Fig 3 right graph in each panel) showed that for most features (*n* = 60 for pyramidal cells and *n* = 47 for *cAC* interneurons, see S1–S4 Tables) the associated error was below 2 sd. Similar results were obtained for the optimizations of *bAC* and *cNAC* interneurons (see individual optimization files at https://collab.humanbrainproject.eu/#/collab/18565). Taken together, these results show that the overall optimization process is a robust way to obtain a number of biophysically accurate neuron models of hippocampal CA1 pyramidal cells and interneurons, which are able to reproduce many of the properties observed experimentally in different types of neurons.

**Fig 4:**
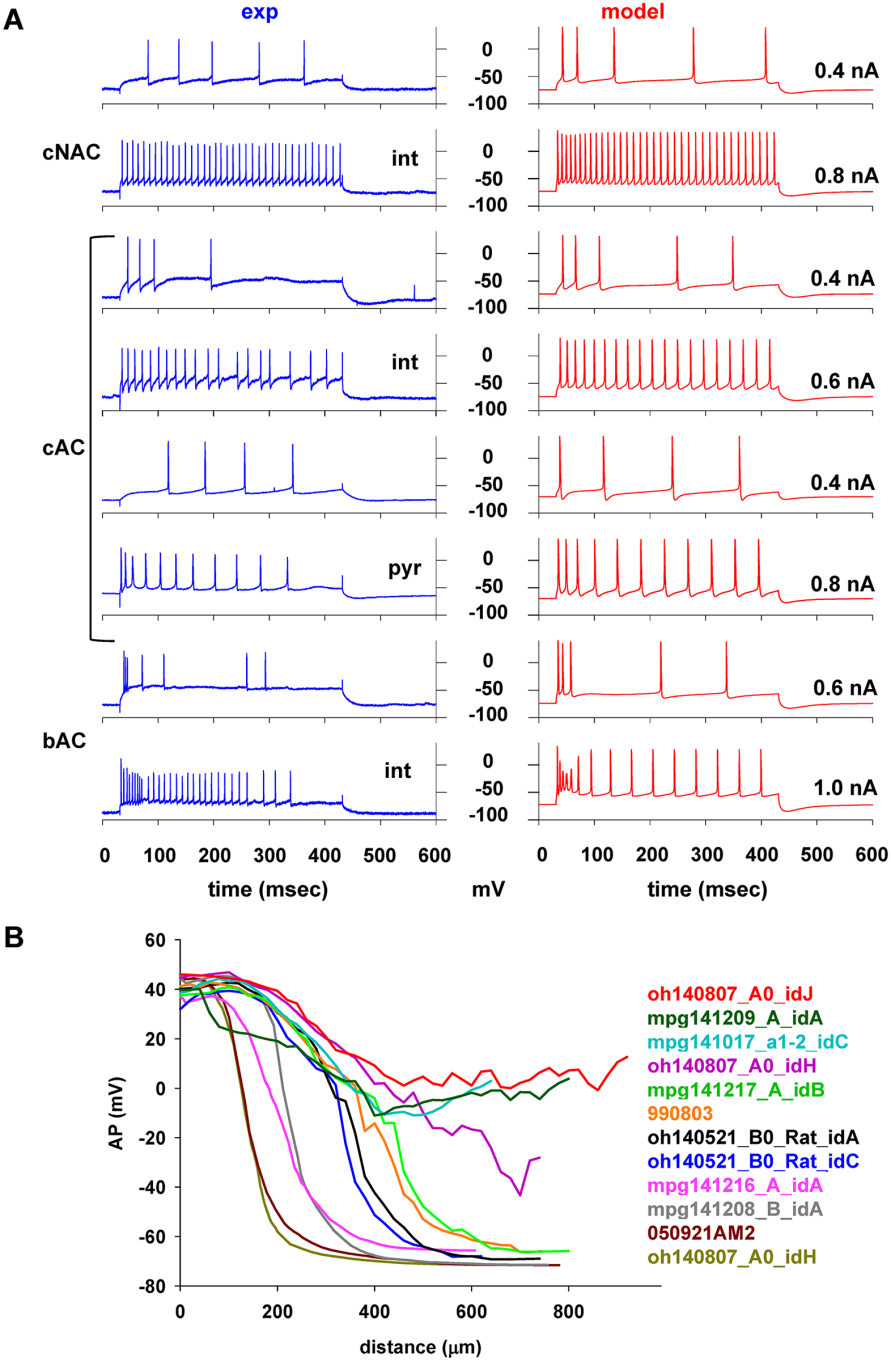
Optimization results. (A) Comparison between typical experimental and model traces for each e-types under different somatic current injection. (B) Peak amplitude of an AP backpropagating in the main apical dendritic trunk of different pyramidal cell models, as a function of the distance from the soma. Each trace refers to a different morphology, as indicated. Abbreviations: *cAC*, continuous accommodating cells; *cAC*, bursting accommodating cells; *cNAC*, continuous non-accommodating cells.

A more direct comparison between experimental and modeling traces for the different e-types is shown in Fig 4A, revealing a very good qualitative agreement between the modeling results and experimental traces. The optimization enabled the production of models that correctly reproduced many characteristics of the firing patterns, such as the strong accommodation observed in *cAC* interneurons (Fig 4A, *cAC* int @0.4nA), the high firing frequency of *bAC* interneurons at the beginning of a current injection (Fig 4A, *bAC* @0.6nA), and the progressive reduction in the AP amplitude during the first part of stronger stimuli (Fig 4A, *bAC* @1nA). The pyramidal cell models also exhibited a typical property often observed experimentally in this type of cells, i.e. the decrease in the peak amplitude of an AP backpropagating into the apical dendrites [20]. This effect has been shown to depend on the high density of A-type potassium channel in the apical dendrites [21], but not all CA1 pyramidal neurons exhibit this effect [22, 23]. It is important to note that this feature was not used to constrain the optimization but, interestingly, the optimized models were able to reproduce it, as shown in Fig 4B, for a few cases using morphologies from both young adult (cells 050921AM2, and 990803) and P14-23 animals. The dichotomy in AP backpropagation observed in the experiments [22] was also reproduced by the model neurons, with the AP amplitude either strongly decreasing beyond ~150 μm from the soma or limited to ~50% of the maximum, with very few cases in between. Taken together, this comparison between experiments and models at the individual trace level, suggests that the optimization process was able to correctly capture and explain both intra- and inter-cell variability in firing behavior in terms of different combinations of active and passive membrane properties.

**Fig 5:**
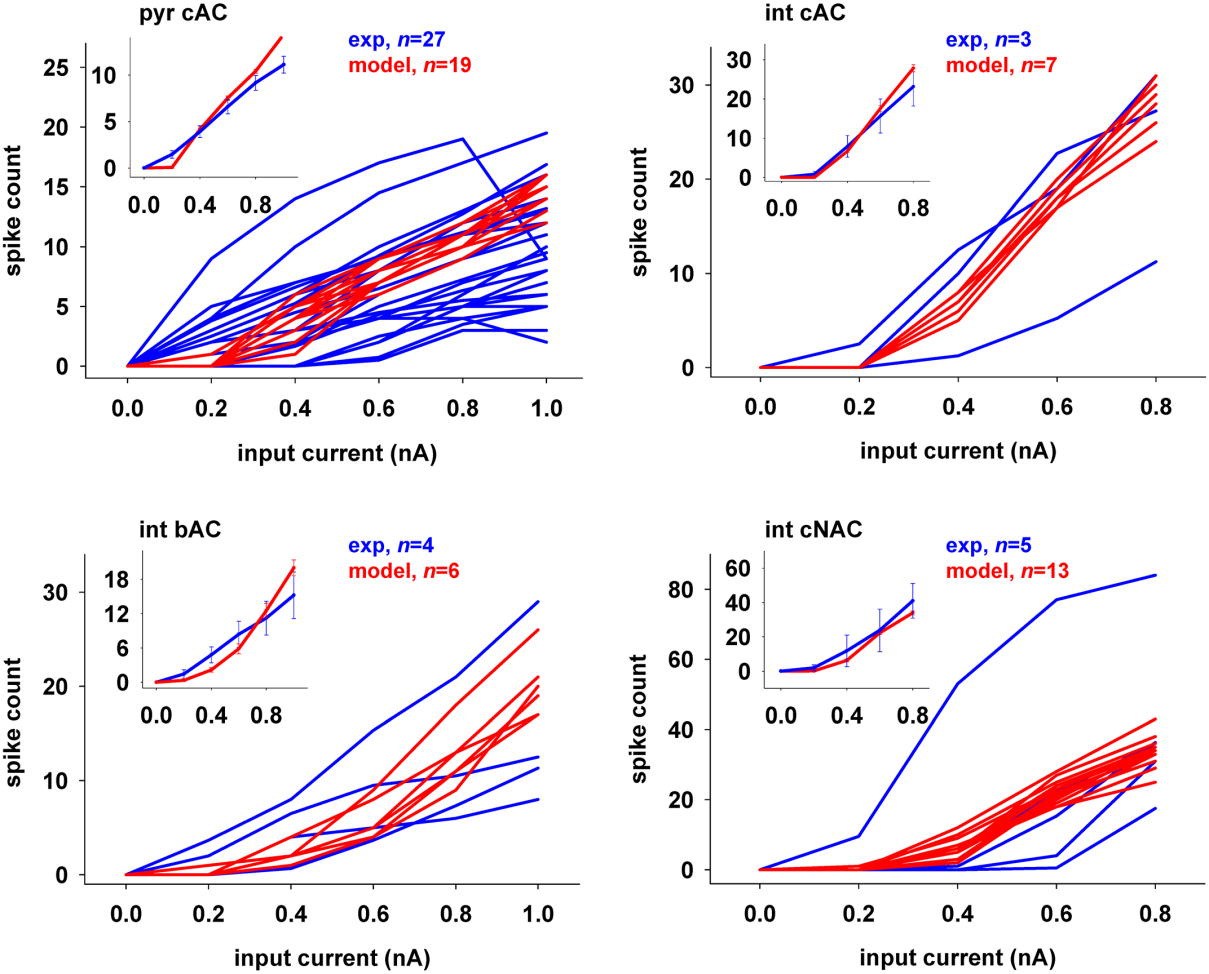
Input/Output properties. Number of spikes as a function of the input current from experiments (blue traces) and models (red traces) for the various e-types. The insets show the corresponding average values. Abbreviations as in Fig 4.

An indication of how the optimized models may capture the variety of experimental input/output properties can be drawn from Fig 5, where the number of spikes for each e-type was plotted against the somatic current injection, for experimental (blue lines) and modeling traces (red lines). In all cases, experimental traces exhibited a rather large inter-cell variability in the number of spikes elicited by any given input current. It is quite common to see up to a ~5-fold difference in the number of spikes elicited in different cells under the same current injection. In most cases, the models were in quantitative agreement with the average number of spikes generated as a function of the input current (Fig 5, insets, Mann Whitney Rank Sum test *p>*0.05 in all cases except for 1nA injection in pyramidal neurons).

### Degeneracy within a population

With the set of data-driven neuron models obtained for each e-type, we can now analyze how different combinations of peak conductances can result in models able to reproduce equally well the firing properties observed experimentally under different current injection steps. The optimization process generates many of these models (termed “individuals”) because of ion channel degeneracy [5]. As discussed in the *Introduction*, this phenomenon is thought to allow a neuron to adjust its firing properties in a robust and tunable manner.

**Fig 6:**
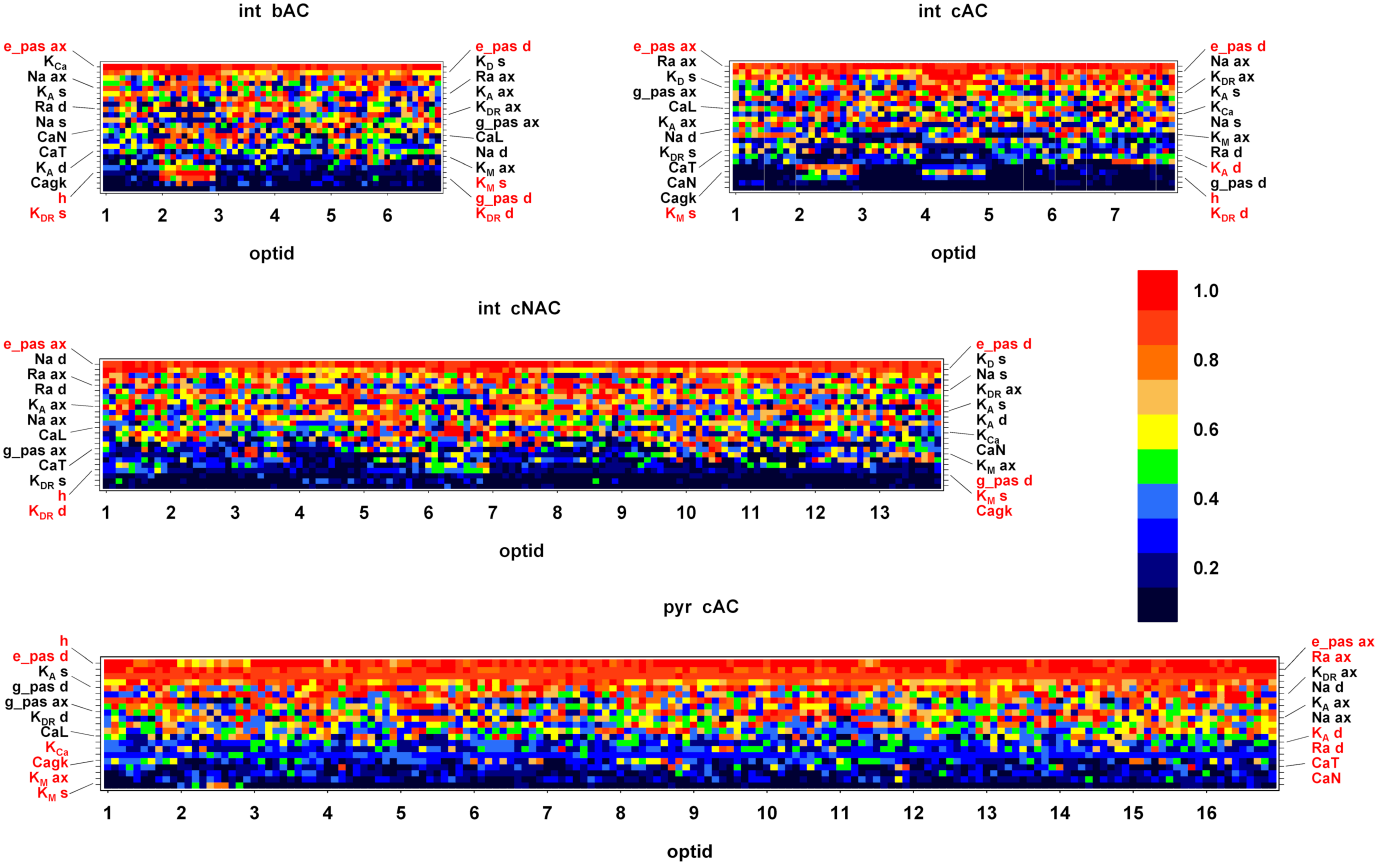
Degeneracy in CA1 pyramidal neurons. Optimized values for all parameters, obtained for the 10 best individuals from each optimization. The X-axis represents the individual optimizations (each composed by 10 individuals), the Y-axis is the parameter’s name. The pixel colors represent the value of the parameter, normalized to the maximum value obtained from all optimizations of a given e-type. The color scale is shown on the right. Abbreviations as in Fig 4. In all cases the total error was in the range of 29–42 sd.

To obtain further insight into on how degeneracy is achieved in hippocampal CA1 neurons, we analyzed all the individuals obtained from the optimization runs. For each optimization run, the 10 best individuals were considered based on their total score (see Methods). Note that these individuals were obtained from the same morphology with different channel densities. In Fig 6, the value of the optimized parameters, normalized to the maximum value chosen for each conductance, were plotted for each optimization run (10 individuals for each run, *opt id*). For clarity, in each graph the values obtained for any given parameter were placed on the Y-axis according to the corresponding average value calculated from all optimizations. In this way, the bottom rows in each graph correspond to parameters with an average low value whereas top rows correspond to parameters with higher values. Furthermore, parameters that were relatively stable across all optimizations (i.e. with a sd<0.2) for any given e-type are highlighted using a red label in the y axis. For pyramidal cells (Fig 6, *pyr cAC*) the most stable parameters were some of the passive properties, I_h_, K_M_, Calcium, and Ca-dependent K currents. Interestingly, we noted that whereas passive properties were consistently optimized with a stable value across the optimizations for all e-types (Fig 6, see top rows in all graphs), conductances were shown to be somewhat different depending on e-types. For example, for interneurons, I_h_, somatic K_M_ and dendritic K_DR_ were the most stable for all e-types, whereas dendritic K_A_ was stable for *cAC* and Cagk for *cNAC*. These results suggested that each e-type has specific active properties that may be particularly important to obtain the appropriate firing pattern in response to a given input. While these properties need to be well constrained for each e-type, degeneracy can be achieved by combining the other conductances in a relatively large number of ways. The functional consequences of this situation will be discussed below.

**Fig 7:**
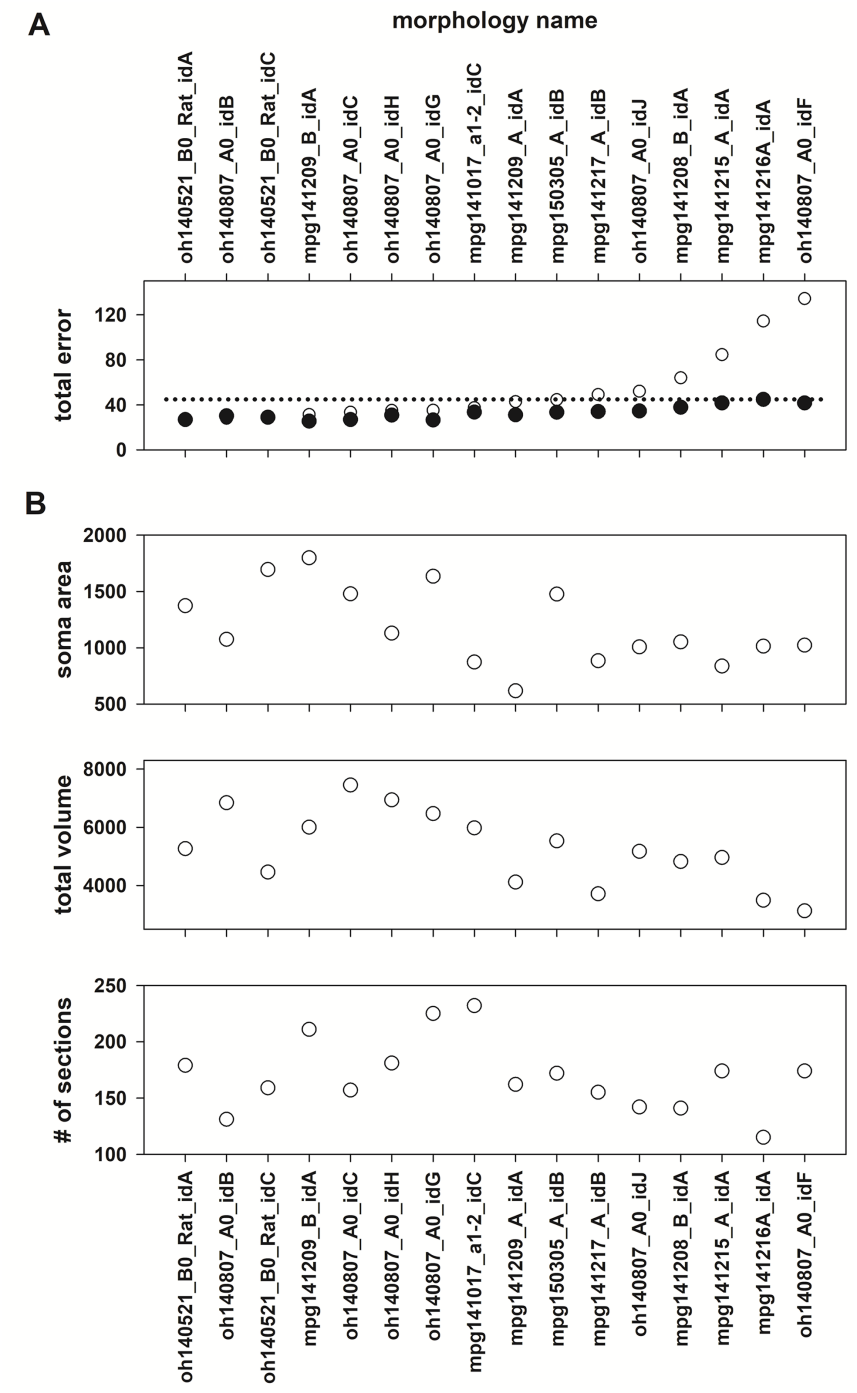
Degeneracy from different morphologies. (A) (*Black symbols*): the total error calculated from the best individual obtained for each morphology; the dotted line identifies the maximum total error. (*Open symbols*): total error calculated from all morphologies equipped with the set of conductances obtained for oh140521_B0_Rat_idA. (B) Soma area, total cell volume, and number of sections of all morphologies.

To explore whether a cell’s morphology can also be related to degeneracy, we fixed the peak conductance values to those found for the best overall individual (obtained for morphology oh140521_B0_Rat_idA) and calculated the total error by using different morphologies. The results are shown in Fig 7A. We found that the total error using the same set of conductances on different morphologies was within the range obtained for each cell’s optimization for 10 out of 16 morphologies. For these cases, there was no correlation between the total error and the main morphological properties, such as soma area, total cell volume, or number of sections (Fig 7B, Spearman correlation, *p*>0.05 in all cases). These results suggest that degeneracy can also be obtained using different morphologies equipped with identical peak channels conductance. A deeper analysis of this issue however was not further considered in this work.

**Fig 8:**
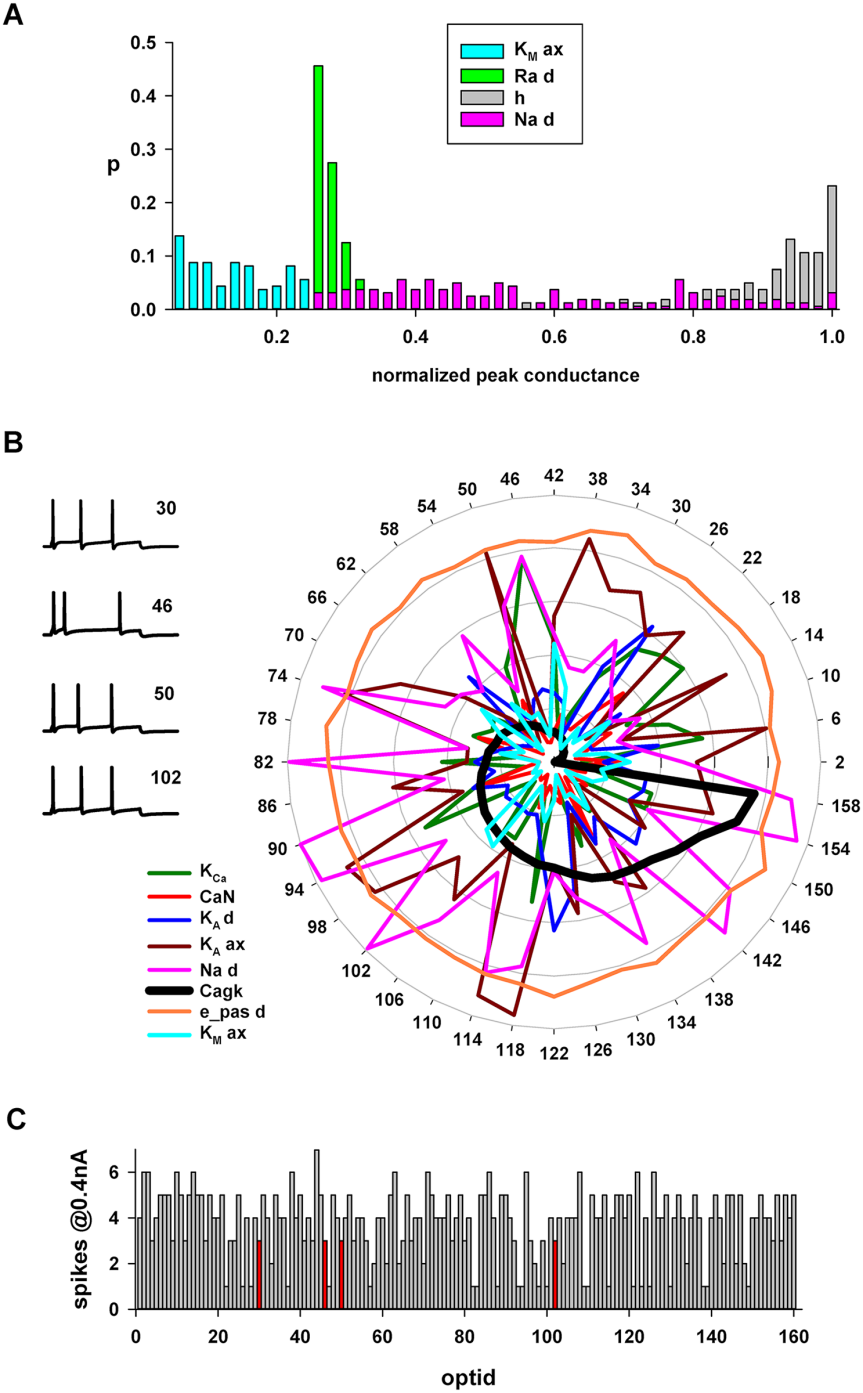
Degeneracy in CA1 pyramidal neurons. (A) Distribution of the normalized values obtained for the somatic K_M_, dendritic Na, I_h_ and Ra. (B) Radar plot with the values obtained for a subset of conductances. Parameters’ values were sorted for those obtained for Cagk (black line); Traces on the left are model traces from individuals #30, 46, 50 and 102 under a 0.4 nA somatic current injection. (C) Number of spikes elicited by a 0.4 nA current injection in each individual. Abbreviations as in Fig 4.

For a more detailed analysis of the configuration of peak conductance values for all models, we first considered the results for pyramidal neurons. In Fig 8A we show a typical distribution of normalized values obtained for membrane properties where optimizations yielded a relatively narrow range (somatic K_M_, I_h_, and Ra), or a wider range of values across individuals (dendritic Na). Note that two of the conductances with a narrow distribution are, in pyramidal CA1 neurons, the dominant factors in controlling major properties such as excitability and accommodation (K_M_, reviewed in [24]), and synaptic integration (I_h_, [25]). The paramount importance of these two types of conductance for reproducing the experimental traces, suggested by their value lying in a narrow range across individuals, emerged from the optimization process without any specific constraint.

An insight on degeneracy in these neurons can be obtained by considering correlation between parameter pairs. In most cases, we found no statistically significant correlation (see S6 Table for the Spearman correlation coefficients). However, for several cases a significant correlation between selected parameters was found (S6 Table, grey cells). The conductance which was most correlated with others was Cagk, a Ca- and voltage-dependent K^+^ conductance that is one of the major determinants for accommodation in these neurons. The inverse correlation with the K_M_ is particularly interesting, since it supports the experimental finding that these channels operate in combination to control intrinsic hyperexcitability [26], and modeling results suggesting how they must both be involved to obtain a strong accommodation [27, 28].

To explore the configuration of the conductances in a more qualitative and intuitive way, we arranged a radar plot of the conductances most correlated with Cagk (Fig 8B), and one of those showing little variability (in this case the reversal potential of the leakage current in the dendrites, e_pas d). The different individuals were sorted with respect to Cagk (Fig 8B, thick black line) and, for clarity, we plotted only 40 of the 160 individuals. The highly jagged and intermixed lines represent the different peak conductance type and value for different individuals giving equally good representations of 60 electrophysiological features experimentally observed in these neurons (see S1 Table). Examples of model traces from a few individuals (all obtained with a 0.4nA somatic current injection) displayed the same number of spikes obtained with very different channel configurations. The number of spikes elicited for each individual is plotted in Fig 8C.

These results give a clear indication that degeneracy in CA1 pyramidal cells can easily emerge from many different combinations of many, but not all, channels. The reason for the lack of pairwise correlation between most parameters does not exclude that the parameter space may be shaped by higher order correlations that can be ultimately responsible for degeneracy. However, a full quantitative study of higher order correlations was outside the scope of this study.

**Fig 9:**
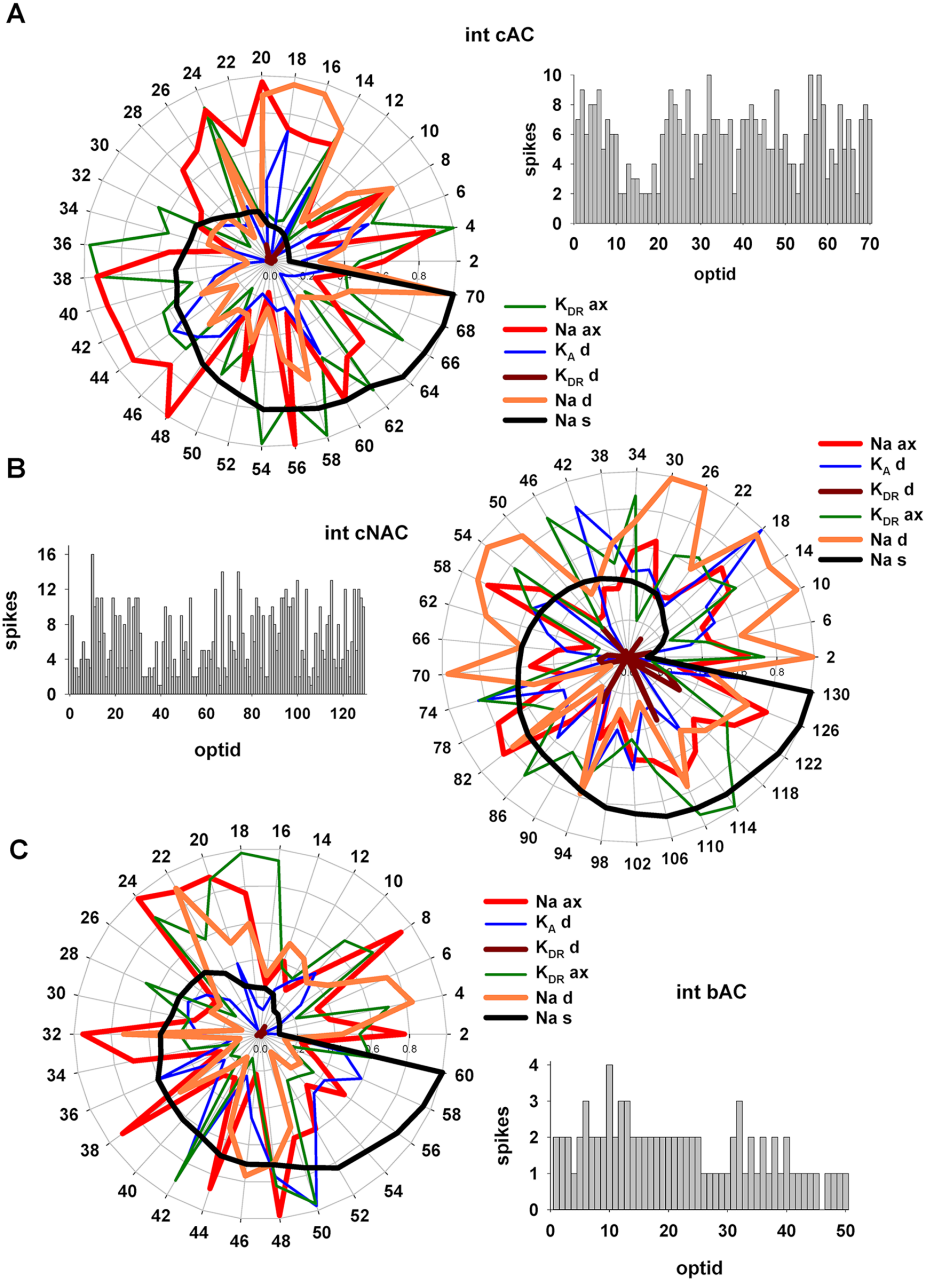
Degeneracy in CA1 interneurons. Radar plots with the values obtained for a subset of conductances. Parameters were sorted for the somatic Na values (black line); the bar graph on the right of each radar plot represents the corresponding spike count from each individual.

The results obtained for interneurons are shown in Fig 9. In this case, to allow an easier comparison of the parameters among the different e-types, individuals were sorted according to the somatic Na conductance (Fig 9, thick black lines), which was among the most correlated with all the others (see S7–S9 Tables). The models suggest a few distinct differences among the different e-types. Note, for example, the distribution of values obtained for the peak conductance of dendritic K_DR_ or K_A_ in the various e-types (Fig 9, dark red and blue lines, respectively), or the difference in the overall values of dendritic Na (Fig 9, orange lines) between *cAC* and *cNAC*. In general, however, the distribution of values were analogous to those obtained for pyramidal cells, with each individual characterized by a highly variable combination of values for many conductances.

### Differences in channel proportions among hippocampal CA1 e-types

**Fig 10:**
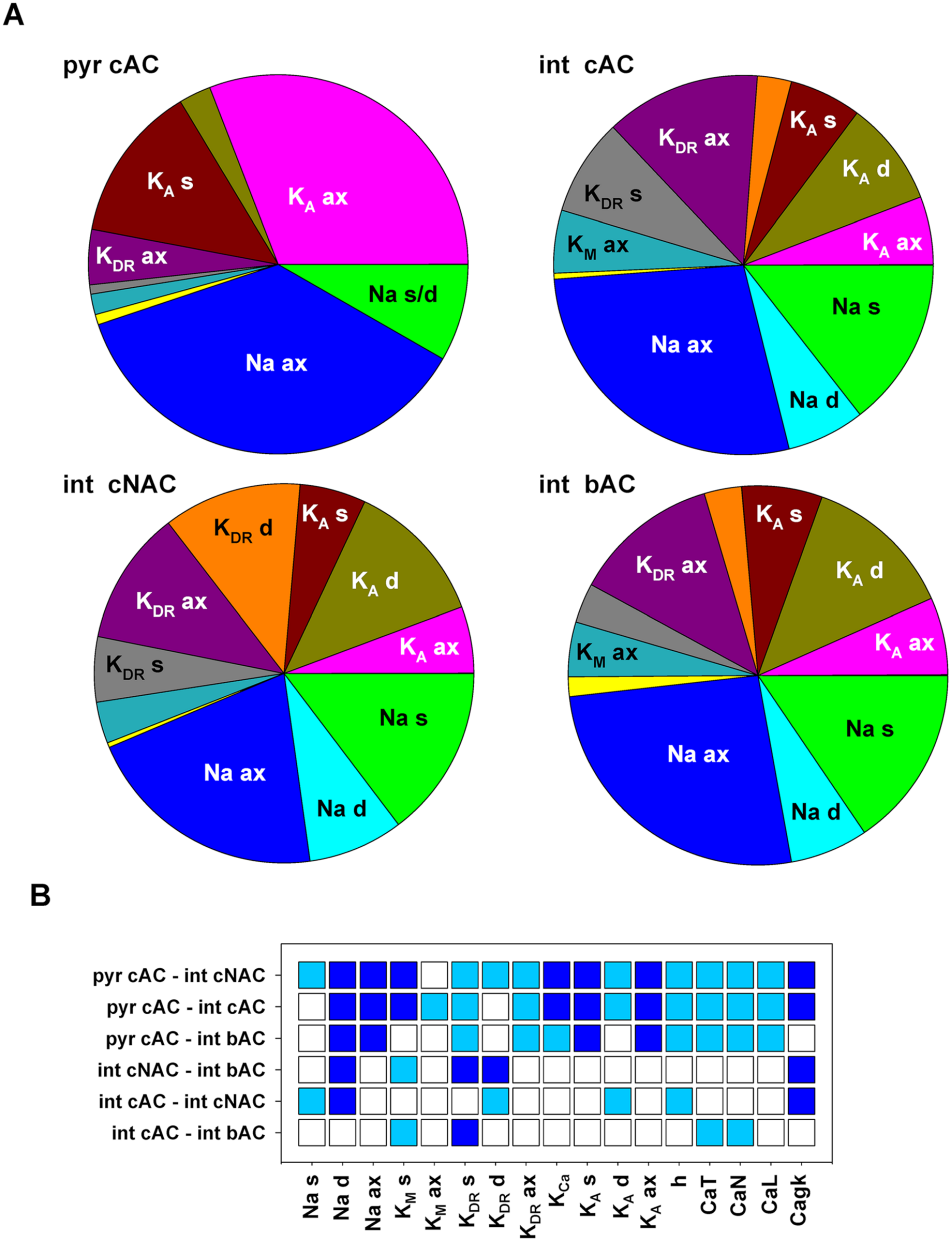
Differences among CA1 neuron populations. (A) Pie charts showing for the different e-types the proportion of each conductance with respect to the total average peak conductance calculated across all individuals. (B) Schematic representation of a Pairwise Multiple Comparison Procedure (Dunn’s Method), between each pair of e-types. The colored boxes indicate cases for which *p*<0.050. Dark blue or cyan indicates that the average value of the first component is significantly lower or higher, respectively, than the second one. An empty box indicates no statistically significant difference.

Finally, one important factor in determining the firing characteristics of different neurons, in addition to a substantial change in morphology [29] and/or gene expression profile [30], is the relative proportion with which specific channels are expressed on the membrane. For this reason, from the optimized models we calculated the relative contribution of each channel in each e-type, by considering the average value of each peak conductance calculated across all individuals. The results are presented in Fig 10A. In all cases, we found that Na, K_A_ and K_DR_ could account for most of the channels expressed on the membrane. Interestingly, each e-type showed a distinct proportion of these channels, with axonal Na channels playing a relatively large role in all e-types, axonal K_A_ being relatively more important in pyramidal neurons than in interneurons, and dendritic K_DR_ being significantly higher in *cNAC* e-types. An analysis of the relative level of each conductance in the various e-types (Fig 10B) also showed significant differences in several cases (Pairwise Multiple Comparison Procedure, p<0.05). From the results it is clear, for example, that dendritic Na should be higher in pyramidal cells than in any type of interneuron, *cAC* interneurons should have a higher dendritic Na among interneurons (Fig 10B, dark blue squares for Na d), and that the axonal K_M_ is essentially independent from cell type. In summary, these results suggest the experimentally testable prediction that different e-types can be characterized by a different combination of the same set of conductances.

## Discussion

It has been shown that any individual neuron can express a distinct combination of many channel types [30] determining its electrical properties [31]. Furthermore, several seminal papers demonstrated that each cell type could exhibit specific correlation between channels expression [32], which may emerge from a homeostatic rule [2]. The overall picture is one in which many different conductances coincide to produce the electrophysiological patterns that characterize the operating range of any given population of neurons, and they do so in such a way to compensate for relatively large changes in individual channel density or synaptic connectivity [33]. The robustness of this mechanism relies on degeneracy [4], which can be practically implemented through a large and flat parameter space for channel conductance. This issue has been studied in the crab pyloric neurons [3], stomatogastric ganglion neurons (e.g. [2, 6]), in the Globus Pallidus neurons of the rat [7]. The presence of degeneracy had yet to be studied in hippocampal neurons. Two recent modeling studies, in the mouse corticospinal neurons and motor cortex, have explicitly shown how degeneracy in cortical neurons can work to implement some electrophysiological features but not others [34], and that degeneracy can also generate multitarget routes from pathological to physiological network dynamics [35]. The first finding was particularly relevant for our study, and it was among the reasons why we choose not to include the voltage between spikes among the optimized features. Its accurate reproduction would have required us to additionally optimize channel kinetics, which was not within the scope of this work.

The analysis of the modeling results presented in this paper provides many experimentally testable predictions on the possible co-regulation of ion currents in hippocampal CA1 neurons. Correlation between pairs of specific conductances has been found for cells in the stomatogastric ganglion of the crab (STG, [32]) and in the pyloric network of the spiny lobster [36]. These experiments found that several pairwise correlations between the same conductances can be present in different type of cells, but no cell type showed conductances with the same set of pairwise correlations. Our optimized models confirmed this result also for the hippocampal CA1 neurons. The models also confirmed pairwise correlations already observed in STG, such as that between K_A_ and I_h_, Na, K_DR_, and Cagk, and between Na and Cagk. Like in the STG, these correlations were observed in different combinations among different cell types. It is important to stress that the optimization process did not bias the parameter values against each other. Correlations thus emerged naturally from the optimization process, and reflected a better reproduction of the experimental features. The models predict several additional pairwise correlations between conductances (see S6–S9 Tables), which are specific for each e-type. All predictions can be tested experimentally, by directly measuring and comparing peak ion currents or (better) channel densities in different neurons or by a genetic perturbation of channel expression [36, 37].

A limitation of this work is that the optimization process was not able to generate a population of models reproducing the very large experimental variability. The reason for this effect is that, in this work, we choose to optimize the different e-types using for each feature the average and standard deviation calculated from all traces, rather than independently optimizing models constrained by traces from an individual cell. A partial explanation for this choice was the limited availability of experimental data on individual cells. Nevertheless, we think that these results offer a significant improvement on the current state of the art, and a necessary step towards building a full-scale cellular model of the rat hippocampus CA1 circuit (Romani et al., in preparation).

Another experimentally testable prediction of the models is that each type of cell should have a small number of channel types that would be expressed at the same density in the same neuronal population. There is already some experimental indication that this is the case for STG cells in the crab [1], where it has been found that K_DR_ is relatively constant among the lateral pyloric neurons of different animals, whereas K_A_ and Cagk varied more than threefold. In this study, we found that passive properties, K_M_, and I_h_ were among the most stable intrinsic membrane properties in any given neuron population, together with dendritic K_DR_ for interneurons.

The models also predict that a different combination of axosomatic Na, K_A_, and K_DR_ channels may dominate the distribution of channels on the membrane of a neuron belonging to a given e-type. This is also experimentally testable, by directly measuring the density of the different channels expressed on the membrane of different type of neurons.

Our analysis suggests a physiological plausible explanation for why single channel mutations can have more or less pathological consequences. A clear example stands out for K_M_ and I_h_ channels in pyramidal cells. We found that these channels must be expressed with a relatively stable density; they do not appear to contribute to degeneracy. This may explain why specific mutations of K_M_ channels can result in neonatal epilepsies in humans [38], or why the decrease in I_h_ caused by experimental models for temporal lobe epilepsy can result in major changes in the electrophysiological mechanisms related to cognitive functions [39].

Finally, the modeling effort presented and discussed in this work is part of a larger modeling workflow currently underway in the framework of the EU Human Brain Project (https://www.humanbrainproject.eu/en/), with the main goal to implement a cellular data-driven model of the entire hippocampus. The Hippocampus is a complex brain structure, deeply embedded into the temporal lobe, with a paramount importance for higher brain functions such as learning and memory, and spatial navigation, and is involved in several major brain diseases. In spite of intensive experimental and computational studies, the mechanisms underlying these functions (and dysfunctions) are still poorly understood. A model implementation and analysis at the cellular level may pave the way for a deeper understanding of the diverse and complex functions of this brain region, and of its levels of organization. One of the major steps towards this goal is the implementation of morphologically and biophysically accurate single cell models for the main neuronal populations, equipped with a set of axonal, somatic, and dendritic currents consistent with many experimentally measured electrophysiological features, in such a way as to be able to capture the main I/O properties observed experimentally. Here we have used a general, robust, and flexible tool able to produce, using reasonable computational resources, ensembles of this type of models for CA1 pyramidal cells and interneurons.

## Methods

### Experimental procedures for interneurons and pyramidal cells 050921AM2, and 990803

#### Electrophysiology

All procedures used throughout this study were carried out according to the British Home Office regulations with regard to the Animal Scientific Procedures Act 1986. Hippocampal slices were prepared as described previously [40, 41]. Briefly, young adult male rats (Sprague-Dawley, body weight 90–180 g) were deeply anaesthetised with Fluothane (inhalation) and sodium pentobarbitone (Sagatal, 60 mg kg^-1^, Rhône Mérieux, Harlow, UK) and perfused transcardially with ice-cold modified artificial cerebrospinal fluid) containing in mM: 248 Sucrose, 25.5 NaHCO_3_, 3.3 KCl, 1.2 KH_2_PO_4_, 1 MgSO_4_, 2.5 CaCl_2_, 15 D-Glucose, equilibrated with 95% O_2_/5% CO_2_. 450 to 500 μm coronal sections were cut (Vibroslice, Camden Instrument, Loughborough, UK) and transferred to an interface recording chamber. They were maintained in modified ACSF solution for 1 hour, and then in standard ASCF (in mM: 124 NaCl, 25.5 NaHCO_3_, 3.3 KCl, 1.2 KH_2_PO_4_, 1 MgSO_4_, 2.5 CaCl_2_, and 15 D-glucose, equilibrated with 95% O_2_/5% CO_2_) for another hour at 34–36°C before commencing electrophysiological recordings. Intracellular recordings were made using sharp microelectrodes (tip resistance, 90–190 MΩ) filled with 2% biocytin in 2M KMeSO4 under current-clamp (Axoprobe; Molecular Devices, Palo Alto, CA). Current-voltage characteristics of CA1 pyramidal cells and interneurons were obtained from their responses to 400 ms current pulses and recorded with pClamp software (Axon Instruments, USA). Individual neurons were recorded and biocytin-filled for up to 3 hours.

#### Histology

The histological procedures have been described previously [42]. Briefly, the 450–500 μm slices were fixed overnight (4% paraformaldehyde (PFA), 0.2% saturated picric acid solution, 0.025% glutaraldehyde solution in 0.1 M Phosphate buffer). Slices were then washed, gelatin-embedded and 50–60 μm sections were cut. Sections were cryoprotected with sucrose, freeze-thawed, incubated first in ABC (Vector laboratories) and then in DAB (3, 3' diaminobenzidine, Sigma) to visualize the biocytin and reveal the morphology of the recorded neurones. Sections were then post-fixed in Osmium Tetroxide, dehydrated, mounted on slides (Durcupan epoxy resin, Sigma) and cured for 48 h at 56°C. The calcium-binding protein and peptide content of some interneurons was investigated by immunofluorescence. Sections were cut and permeabilised with sucrose and freeze-thawed. They were then incubated in 1% Sodium Borohydride (NaBH_4_) for 30 minutes, in 10% normal goat serum for another 30 min and then incubated overnight in a primary antibody solution (mouse monoclonal anti-Parvalbumin (Sigma) or rabbit polyclonal anti-calbindin (CB) (Baimbridge & Miller, 1982)) made up in ABC solution. Sections were then incubated for 2h in a solution of fluorescently labelled secondary antibodies (anti-mouse fluorescein isothiocyanate (FITC) and/or goat anti-rabbit Texas Red (TR), and Avidin-7-Amino-4-methylcoumarin-3-acetic acid (Avidin-AMCA) made up in PBS). Sections were mounted on slides in Vectashield (Vector laboratories) and studied by fluorescence microscopy. Subsequently, sections were incubated in ABC (Vector laboratories) and then in DAB (3, 3' diaminobenzidine, Sigma) to visualise the biocytin, post-fixed, dehydrated, mounted on slides and cured for 48 h at 56°C. All CA1 neurons were then reconstructed using a Neurolucida software (MBF Bioscience).

#### Histological procedures for pyramidal cells, except cells 050921AM2, and 990803

For all the other pyramidal cells, *ex-vivo* coronal preparations (300 μm thick) were obtained for the hippocampus of wild type rats (Wistar) brains, post-natal 14–23 days. The project was approved by the Swiss Cantonal Veterinary Office following its ethical review by the State Committee for Animal Experimentation. All procedures were conducted in conformity with the Swiss Welfare Act and the Swiss National Institutional Guidelines on Animal Experimentation for the ethical use of animals. All *ex-vivo* brain slices were cut in ice-cold aCSF (artificial cerebro-spinal fluid) with low Ca^2+^ and high Mg^2+^. The intracellular pipette solution contained (in mM) 110 Potassium Gluconate, 10 KCl, 4 ATP-Mg, 10 Phosphocreatine, 0.3 GTP, 10 HEPES and 13 Biocytin, adjusted to 290–300 mOsm/Lt with D-Mannitol (25–35 mM) at pH 7.3. Chemicals were from Sigma Aldrich (Stenheim, Germany) or Merck (Darmstadt, Germany). A few somatic whole cell recordings (not available for this work) were performed with Axopatch 200B amplifiers in current clamp mode at 34 ± 1°C bath temperature. After the recordings, cells were left in whole cell mode for 45mins for biocytin to fill up the cell. The pipette was then carefully removed and the brain slice placed in PFA 4% overnight. Slice were then placed in PBS 1X, biocytin revealing protocol was performed prior to mounting. Reconstruction made by eye with assistance of camera Lucida.

#### Computational methods

The models have been implemented using three-dimensional morphological reconstructions. Electrophysiological features of interest (see next paragraph) were extracted from experimental traces using custom code exploiting the open source Electrophysiological Feature Extraction Library (eFEL, https://github.com/BlueBrain/eFEL). Extracted features were then used for multi-objective model parameter optimizations performed using the open source Blue Brain Python Optimization Library (BluePyOpt, [43]). Both are part of a set of tools integrated into many online use cases of the Brain Simulation Platform (BSP) of the Human Brain Project (https://www.humanbrainproject.eu/en/brain-simulation/). The optimizations were carried out using HPC systems, accessible from the BSP, at either the Neuroscience Gateway (https://www.nsgportal.org/), CINECA (Bologna, Italy), or JSC (Jülich, Germany). On a KNL-based HPC system, a typical optimization run for a pyramidal cell, configured to generate 128 individuals/generation, required approximately 1 hour/generation using 128 cores. Typical production runs for each optimization required approximately 60 generations to reach an equilibrated state.

The overall optimization approach, of using a genetic algorithm, was similar to other studies (e.g. [35, 44]), but with important qualitative differences: for example, in [35] only one detailed morphology was used, whereas in [44] the authors tested many detailed morphologies but with the soma as the only active compartment. In our case, we used many detailed morphologies and, in all of them, we distributed dendritic conductances constrained by experimental findings. This allowed us, for example, to also reproduce experimental dendritic recordings. We believe that for studying degeneracy of ionic currents in hippocampal pyramidal neurons, known to have active dendrites with fundamental roles in signal integration, our choice can give better results.

All experimental and model files are publicly available at the Human Brain Project collab https://collab.humanbrainproject.eu/#/collab/18565. Complete model and simulation files will also be available on the ModelDB section of the Senselab suite (https://senselab.med.yale.edu/modeldb/ acc. n.244688).

Readers interested in running their own optimization can also access the public “Online Use Cases” of the BSP directly related to single cell modeling (https://collab.humanbrainproject.eu/#/collab/1655/nav/28538). A number of tools with an intuitive graphical user interface will guide the user through all steps, from selecting experimental data to constrain the model, to running an optimization to generate a model template and, finally, to exploring the model with *in silico* experiments.

#### Electrophysiological features

Thousands of electrophysiological features may be used to constrain a model’s optimization process and many hundreds of parameters to optimize. Ideally, all of them should be used. In practice, however, this is essentially impossible. The amount of missing information will make the problem ill-defined, and the sheer number of parameters that would be required will result in a substantial overfitting. For this reason, we decided to take into account a selected set of electrophysiological features for each e-type, listed in S1–S4 Tables. They include features that are particularly important in shaping the I/O properties of a neuron, such as the spike count and spike times, and those associated with the resting potential and the input resistance. Their average (±sd) value was calculated from experimental traces, using a custom version of the feature extraction tool.

A total of 225 experimental features were used to constrains the optimization process.

#### Models configuration

Given the experimentally known differences between pyramidal cells and interneurons, we used different channels’ configuration and distribution, as schematically illustrated in S1 Fig. Channel kinetics were based on those used in many previously published papers on hippocampal neurons [45, 46], and validated against a number of experimental findings on CA1 pyramidal neurons. The complete set of active membrane properties included a sodium current (Na), four types of potassium (K_DR_, K_A_, K_M_, and K_D_), three types of Calcium (CaN, CaL, CaT), the nonspecific I_h_ current, and two types of Ca-dependent K^+^ currents, K_Ca_ and Cagk. A simple Calcium extrusion mechanism, with a single exponential decay of 100 ms, was also included in all compartments containing Calcium channels. In general, channels were uniformly distributed in all dendritic compartments except K_A_ and I_h_, which in pyramidal cells are known to increase with distance from the soma [20, 25]. The values for the peak conductance of each channel were independently optimized in each type of compartment (soma, axon, basal and apical dendrites). The parameters’ range, independently for pyramidal cells and interneurons, was defined with preliminary simulations, and it covered a range of at least one order of magnitude.

We realized that one of the sentences in the Discussion “The presence of degeneracy has not been systematically studied in hippocampal neurons yet” may incorrectly convey the notion that there are no previous studies on degeneracy in the hippocampus. We would like to point out that this is not the case. It is worth mentioning that degeneracy has been previously studied in one CA1 pyramidal neuron morphology, to study the emergence of a few functional maps [47], or in the context of spectral tuning (reviewed in [48]) and synaptic integration and plasticity [49] using either a single morphology or a reduced model of CA1 pyramidal neurons.

## Supporting information

S1 Fig CA1 pyramidal neuron and interneuron active properties. Morphologies of a pyramidal neuron (*left*) and an interneuron (*right*), with a schematic indication of channels’ distribution on the soma, axon, and dendrites.
(DOCX)

S1 Table Electrophysiological features used for optimization of pyramidal neurons. (DOCX)

S2 Table Electrophysiological features used for optimization of int *cAC* cells. (DOCX)

S3 Table Electrophysiological features used for optimization of int *bAC* cells. (DOCX)

S4 Table Electrophysiological features used for optimization of int *cNAC* cells. (DOCX)

S5 Table Morphological classes and e-types of the optimized pyramidal cells (*left*) and interneurons (*right*). (DOCX)

S6 Table Spearman correlation coefficient between peak conductance values from pyramidal cell models. Only conductances with at least one significant correlation coefficient >|0.25| (gray cells) are shown. The *p* value corresponding to each coefficient is indicated in italics.
(DOCX)

S7 Table Spearman correlation coefficient between peak conductance values from cNAC interneuron models. Only conductances with at least one significant correlation coefficient >|0.25| (gray cells) are shown. The *p* value corresponding to each coefficient is indicated in italics.
(DOCX)

S8 Table Spearman correlation coefficient between peak conductance values from bAC interneuron models. Only conductances with at least one significant correlation coefficient >|0.25| (gray cells) are shown. The *p* value corresponding to each coefficient is indicated in italics.
(DOCX)

S9 Table Spearman correlation coefficient between peak conductance values from cAC interneuron models. Only conductances with at least one significant correlation coefficient >|0.25| (gray cells) are shown. The *p* value corresponding to each coefficient is indicated in italics.
(DOCX)
