# Supplementary figures and images for: The physiological variability of channel density in hippocampal CA1 pyramidal cells and interneurons explored using a unified data-driven modeling workflow
Source: PLoS Comput Biol. 2018 Sep 17;14(9):e1006423. doi: 10.1371/journal.pcbi.1006423 (PMC6160220; doi:10.1371/journal.pcbi.1006423)

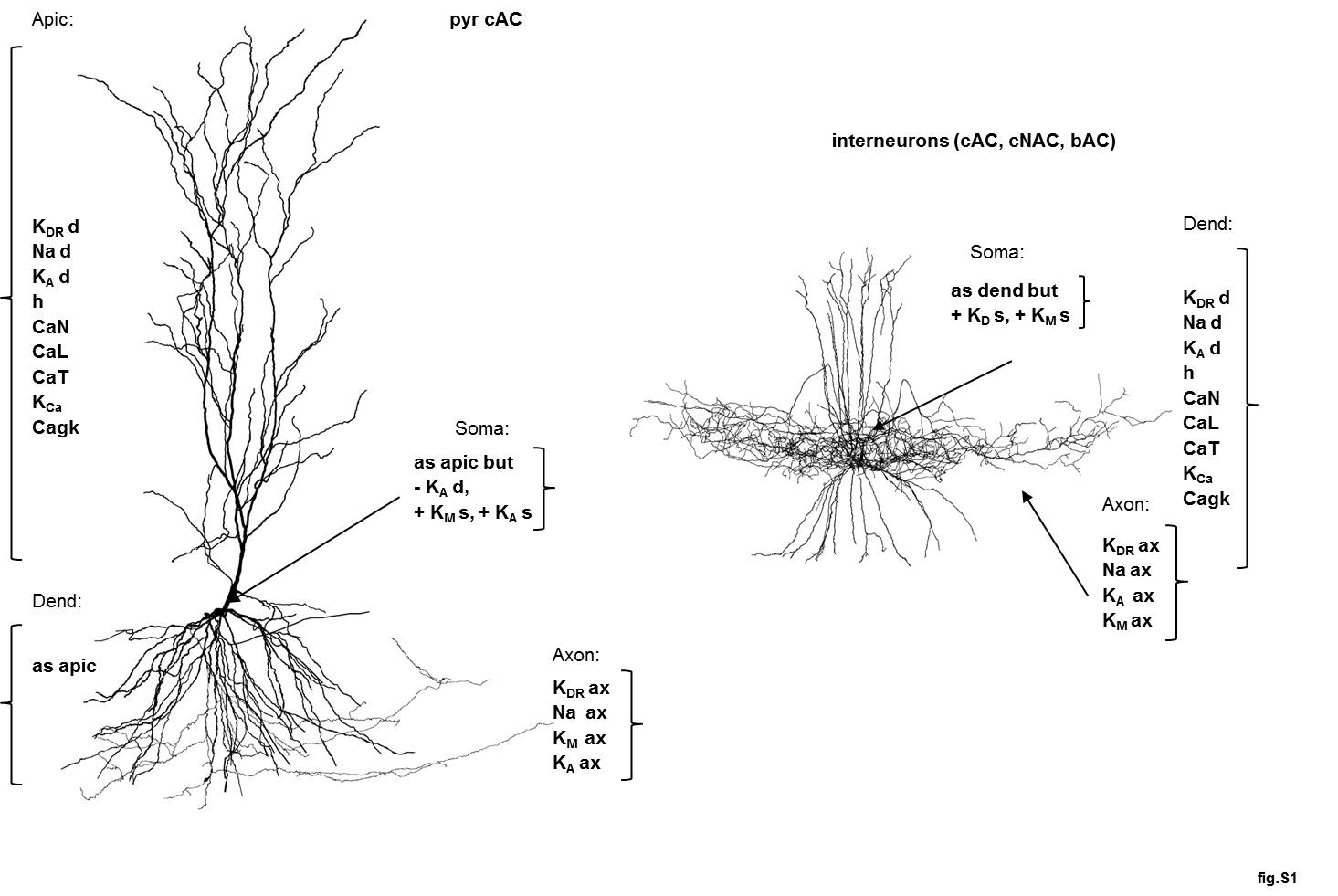

Supplement: S1 Fig — Morphologies of a pyramidal neuron (left) and an interneuron (right), with a schematic indication of channels’ distribution on the soma, axon, and dendrites. (DOCX) [file pcbi.1006423.s001.docx]
