# Supplementary material for: The physiological variability of channel density in hippocampal CA1 pyramidal cells and interneurons explored using a unified data-driven modeling workflow
Source: PLoS Comput Biol. 2018 Sep 17;14(9):e1006423. doi: 10.1371/journal.pcbi.1006423 (PMC6160220; doi:10.1371/journal.pcbi.1006423)
Supplement: S1 Table — (DOCX) [file pcbi.1006423.s002.docx]

| **Feature / Input current** | **-0.8 nA** | **-0.6 nA** | **-0.2 nA** | **0.2 nA** | **0.4 nA** | **0.6 nA** | **0.8 nA** | **1.0 nA** |
| --- | --- | --- | --- | --- | --- | --- | --- | --- |
| **Voltage deflection** | -19.26±4.69 | -14.93±3.52 | -5.59±1.58 |  |  |  |  |  |
| **Voltage base** | -69.59±4.31 | -69.50±4.33 | -69.39±4.31 | -69.23±4.39 | -69.25±4.39 | -69.59±4.36 | -69.74±4.36 | -69.93±4.51 |
| **Spikecount** |  |  |  | 1.46±2.04 | 3.91±3.33 | 6.59±3.98 | 9.17±4.17 | 11.13±4.68 |
| **Time to last spike** |  |  |  | 88.70±125.47 | 198.77±137.73 | 284.51±104.33 | 296.59±90.52 | 302.40±105.88 |
| **Inv time to first spike** |  |  |  | 24.35±33.69 | 61.93±67.20 | 101.75±91.28 | 154.46±117. 99 | 203.69±140.78 |
| **Inv first ISI** |  |  |  | 13.57±23.47 | 40.82±39.86 | 68.38±46.71 | 93.67±45.73 | 115.99±42.56 |
| **Inv second ISI** |  |  |  | 4.45±8.86 | 19.46±21.62 | 41.72±29.40 | 62.64±29.87 | 80.17±30.77 |
| **Inv third ISI** |  |  |  | 2.41±5.74 | 12.19±14.16 | 25.44±19.12 | 39.67±20.46 | 52.87±19.95 |
| **Inv fourth ISI** |  |  |  | 1.23±4.57 | 8.51±12.07 | 17.95±16.93 | 30.38±16.71 | 40.40±15.71 |
| **Inv fifth ISI** |  |  |  | 0.67±3.53 | 6.18±10.21 | 14.21±15.64 | 24.17±18.34 | 33.18±18.13 |
| **Inv last ISI** |  |  |  | 6.07±17.70 | 9.14±8.47 | 13.20±7.63 | 17.53±6.62 | 21.34±7.78 |
| **Mean frequency** |  |  |  |  |  |  | 32.05±10.86 | 38.17±10.00 |
| **Time to first spike** |  |  |  |  |  |  | 13.82±13.28 | 8.50±6.70 |
