# Supplementary material for: The physiological variability of channel density in hippocampal CA1 pyramidal cells and interneurons explored using a unified data-driven modeling workflow
Source: PLoS Comput Biol. 2018 Sep 17;14(9):e1006423. doi: 10.1371/journal.pcbi.1006423 (PMC6160220; doi:10.1371/journal.pcbi.1006423)
Supplement: S2 Table — (DOCX) [file pcbi.1006423.s003.docx]

| **Feature / Input current** | **-0.4 nA** | **-0.2 nA** | **0.2 nA** | **0.4 nA** | **0.6 nA** | **0.8 nA** |
| --- | --- | --- | --- | --- | --- | --- |
| **Voltage deflection** | -19.77±2.03 | -9.86±1.08 |  |  |  |  |
| **Voltage base** | -74.62±2.14 | -74.96±2.87 | -74.85±3.32 | -74.86±3.06 | -74.66±3.31 | -74.62±3.49 |
| **Spikecount** |  |  | 0.83±1.18 | 7.92±4.82 | 15.67±7.52 | 23.17±8.56 |
| **Time to last spike** |  |  | 65.93±93.24 | 276.67±89.38 | 347.74±47.00 | 351.53±29.35 |
| **Inv time to first spike** |  |  | 4.80±6.78 | 72.80±51.42 | 157.46±71.90 | 251.37±64.96 |
| **Inv first ISI** |  |  | 4.30±6.09 | 38.72±25.55 | 75.99±40.95 | 122.74±31.38 |
| **Inv second ISI** |  |  | 1.81±2.56 | 30.74±18.08 | 72.92±29.61 | 113.52±29.38 |
| **Inv third ISI** |  |  |  | 30.74±21.88 | 67.76±28.65 | 109.43±29.03 |
| **Inv fourth ISI** |  |  |  | 21.40±16.11 | 61.18±26.89 | 100.68±30.11 |
| **Inv fifth ISI** |  |  |  | 21.21±16.13 | 53.56±35.23 | 99.49±29.37 |
| **Inv last ISI** |  |  | 3.64±5.14 | 16.60±10.71 | 37.22±2.62 | 59.24±5.78 |
| **Mean frequency** |  |  |  |  | 43.11±16.84 | 65.79±24.72 |
| **Time to first spike** |  |  |  |  | 14.19±12.80 | 5.54±2.77 |
| **AHP_depth** |  |  |  |  | 13.08±2.67 | 19.98±6.48 |
