# Supplementary material for: The physiological variability of channel density in hippocampal CA1 pyramidal cells and interneurons explored using a unified data-driven modeling workflow
Source: PLoS Comput Biol. 2018 Sep 17;14(9):e1006423. doi: 10.1371/journal.pcbi.1006423 (PMC6160220; doi:10.1371/journal.pcbi.1006423)
Supplement: S3 Table — (DOCX) [file pcbi.1006423.s004.docx]

| **Feature / Input current** | **-1.0 nA** | **-0.8 nA** | **-0.6 nA** | **-0.4 nA** | **-0.2 nA** | **0.2 nA** | **0.4 nA** | **0.6 nA** | **0.8 nA** | **1.0 nA** |
| --- | --- | --- | --- | --- | --- | --- | --- | --- | --- | --- |
| **Voltage deflection** | -56.03±3.18 | -42.56±14.52 | -31.97±9.01 | -21.70±5.41 | -11.85±3.45 |  |  |  |  |  |
| **Voltage base** | -72.68±4.02 | -73.57±3.70 | -73.56±4.21 | -73.60±3.57 | -74.00±4.39 | -74.24±4.41 | -74.13±4.50 | -7399±5.10 | 73.85±4.55 | -73.06±3.83 |
| **Steady state voltage** |  |  |  |  | -74.30±3.93 |  |  |  |  |  |
| **Spikecount** |  |  |  |  |  | 1.42±1.53 | 4.79±2.78 | 8.38±4.56 | 11.21±5.88 | 15.21±8.13 |
| **Time to last spike** |  |  |  |  |  | 49.27±50.40 | 22.56±134.54 | 269.17±103.41 | 264.18±80.98 | 328.97±24.83 |
| **Inv time to first spike** |  |  |  |  |  | 29.94±35.83 | 98.46±65.65 | 197.03±97.13 | 215.69±83.21 | 346.55±90.32 |
| **Inv first ISI** |  |  |  |  |  | 12.25±14.36 | 75.68±46.24 | 152.30±92.81 | 214.45±75.16 | 259.14±88.62 |
| **Inv second ISI** |  |  |  |  |  | 10.23±14.37 | 36.15±44.46 | 79.38±37.72 | 156.57±104.06 | 222.50±84.46 |
| **Inv third ISI** |  |  |  |  |  | 7.93±10.91 | 25.59±30.04 | 56.63±52.56 | 105.39±60.22 | 175.40±74.19 |
| **Inv fourth ISI** |  |  |  |  |  | 6.83±11.83 | 31.25±46.17 | 47.99±60.39 | 71.13±50.55 | 110.76±57.73 |
| **Inv fifth ISI** |  |  |  |  |  | 5.63±9.75 | 18.26±25.61 | 40.39±58.13 | 55.43±55.52 | 93.70±79.92 |
| **Inv last ISI** |  |  |  |  |  | 2.54±2.55 | 36.9±36.34 | 64.96±82.22 | 19.71±4.88 | 29.03±12.78 |
| **Mean frequency** |  |  |  |  |  |  |  | 40.84±21.81 | 45.32±14.71 | 46.46±25.84 |
| **Time to first spike** |  |  |  |  |  |  |  | 8.75±6.70 | 5.73±1.98 |  |
| **AHP_depth** |  |  |  |  |  |  |  | 17.97±6.09 | 20.74±5.66 | 21.35±6.15 |
