# Supplementary material for: The physiological variability of channel density in hippocampal CA1 pyramidal cells and interneurons explored using a unified data-driven modeling workflow
Source: PLoS Comput Biol. 2018 Sep 17;14(9):e1006423. doi: 10.1371/journal.pcbi.1006423 (PMC6160220; doi:10.1371/journal.pcbi.1006423)
Supplement: S4 Table — (DOCX) [file pcbi.1006423.s005.docx]

| **Feature / Input current** | **-0.8 nA** | **-0.6 nA** | **-0.4 nA** | **-0.2 nA** | **0.2 nA** | **0.4 nA** | **0.6 nA** | **0.8 nA** |
| --- | --- | --- | --- | --- | --- | --- | --- | --- |
| **Voltage deflection** | -29.63±16.86 | -21.66±12.84 | -15.66±7.65 | -8.74±4.44 |  |  |  |  |
| **Voltage base** | -73.94±4.53 | -74.10±4.58 | -7387±4.54 | -74.10±4.14 | -74.22±4.03 | -74.66±4.559 | -74.67±4.82 | -74.72±5.19 |
| **Spikecount** |  |  |  |  | 1.90±3.80 | 11.80±20.68 | 23.77±27.71 | 40.97±22.57 |
| **Time to last spike** |  |  |  |  | 61.55±123.10 | 164.49±167.42 | 295.55±101.64 | 393.57±5.89 |
| **Inv time to first spike** |  |  |  |  | 15.30±30.60 | 63.50±114.17 | 209.25±211.10 | 341.81±263.40 |
| **Inv first ISI** |  |  |  |  | 3.37±6.73 | 41.51±72.18 | 91.54±94.50 | 140.96±99.55 |
| **Inv second ISI** |  |  |  |  | 16.67±33.34 | 36.43±64.81 | 78.34±81.56 | 127.36±76.02 |
| **Inv third ISI** |  |  |  |  | 2.12±4.25 | 32.69±59.66 | 73.17±79.38 | 124.59±71.75 |
| **Inv fourth ISI** |  |  |  |  | 10.75±21.51 | 33.10±60.20 | 68.48±78.71 | 121.27±69.85 |
| **Inv fifth ISI** |  |  |  |  | 8.47±16.95 | 28.13±56.25 | 65.72±75.50 | 123.25±66.96 |
| **Inv last ISI** |  |  |  |  | 7.64±15.29 | 33.60±59.08 | 55.45±61.49 | 90.66±53.55 |
| **Time to first spike** |  |  |  |  |  |  |  | 9.87±12.70 |
