# Supplementary material for: The physiological variability of channel density in hippocampal CA1 pyramidal cells and interneurons explored using a unified data-driven modeling workflow
Source: PLoS Comput Biol. 2018 Sep 17;14(9):e1006423. doi: 10.1371/journal.pcbi.1006423 (PMC6160220; doi:10.1371/journal.pcbi.1006423)
Supplement: S5 Table — (DOCX) [file pcbi.1006423.s006.docx]

| **cell name** | **m-type** | **cAC** | **bAC** | **cNAC** |  | **cell name** | **m-type** | **cAC** | **bAC** | **cNAC** |
| --- | --- | --- | --- | --- | --- | --- | --- | --- | --- | --- |
| 990803 | SP_PC | ***** |  |  |  | 010710HP2 | SP_Ivy | ***** |  | ***** |
| 050921AM2 | SP_PC | ***** |  |  |  | 011017HP2 | SO_OLM | ***** | ***** | ***** |
| mpg141017_a1-2_idC | SP_PC | ***** |  |  |  | 011023HP2 | SO_BS |  | ***** | ***** |
| mpg141208_B_idA | SP_PC | ***** |  |  |  | 011127HP1 | SLM_PPA |  | ***** |  |
| mpg141209_A_idA | SP_PC | ***** |  |  |  | 031031AM1 | SP_CCKBC | ***** |  |  |
| mpg141209_B_idA | SP_PC | ***** |  |  |  | 060314AM2 | SP_PVBC |  | ***** | ***** |
| mpg141215_A_idA | SP_PC | ***** |  |  |  | 970509HP2 | SO_Tri |  |  | ***** |
| mpg141216_A_idA | SP_PC | ***** |  |  |  | 970627BHP1 | SP_PVBC | ***** |  | ***** |
| mpg141217_A_idB | SP_PC | ***** |  |  |  | 970717D | SP_Ivy |  |  | ***** |
| mpg150305_A_idB | SP_PC | ***** |  |  |  | 970911C | SP_AA |  |  | ***** |
| oh140521_B0_Rat_idA | SP_PC | ***** |  |  |  | 971114B | SO_Tri |  | ***** | ***** |
| oh140521_B0_Rat_idC | SP_PC | ***** |  |  |  | 980120A | SO_BP | ***** | ***** | ***** |
| oh140807_A0_idA | SP_PC | ***** |  |  |  | 980513B | SP_BS | ***** |  | ***** |
| oh140807_A0_idB | SP_PC | ***** |  |  |  | 990111HP2 | SP_PVBC |  |  | ***** |
| oh140807_A0_idC | SP_PC | ***** |  |  |  | 990611HP2 | SR_SCA | ***** |  | ***** |
| oh140807_A0_idF | SP_PC | ***** |  |  |  | 990827IN5HP3 | SR_IS1 | ***** |  |  |
| oh140807_A0_idG | SP_PC | ***** |  |  |  |  |  |  |  |  |
| oh140807_A0_idH | SP_PC | ***** |  |  |  |  |  |  |  |  |
| oh140807_A0_idJ | SP_PC | ***** |  |  |  |  |  |  |  |  |
