# Supplementary material for: The physiological variability of channel density in hippocampal CA1 pyramidal cells and interneurons explored using a unified data-driven modeling workflow
Source: PLoS Comput Biol. 2018 Sep 17;14(9):e1006423. doi: 10.1371/journal.pcbi.1006423 (PMC6160220; doi:10.1371/journal.pcbi.1006423)
Supplement: S6 Table — Only conductances with at least one significant correlation coefficient >|0.25| (gray cells) are shown. The p value corresponding to each coefficient is indicated in italics. (DOCX) [file pcbi.1006423.s007.docx]

|  | **CaT** | **Cagk** | **K_Ca_** | **CaN** | **K_A_ d** | **K_DR_** | **K_A_ ax** | **Na d** | **K_DR_ ax** | **g_pas ax** | **g_pas d** | **Ra ax** | **e_pas d** |
| --- | --- | --- | --- | --- | --- | --- | --- | --- | --- | --- | --- | --- | --- |
| **K_M_ s** | **0.0872** | **-0.302** | **-0.0329** | **0.0513** | **-0.359** | **0.0658** | **0.0299** | **-0.00861** | **-0.0401** | **-0.0781** | **-0.073** | **-0.0306** | **0.153** |
|  | **0.273** | **0.000114** | **0.679** | **0.519** | **0.00000354** | **0.408** | **0.707** | **0.914** | **0.614** | **0.326** | **0.359** | **0.7** | **0.0534** |
|  |  |  |  |  |  |  |  |  |  |  |  |  |  |
| **Cagk** |  |  | **-0.395** | **-0.283** | **0.316** | **0.265** | **-0.286** | **0.27** | **0.0277** | **-0.0786** | **0.16** | **0.174** | **0.13** |
|  |  |  | **0.000000298** | **0.000302** | **0.0000497** | **0.000744** | **0.000265** | **0.000588** | **0.728** | **0.323** | **0.0428** | **0.0282** | **0.101** |
|  |  |  |  |  |  |  |  |  |  |  |  |  |  |
| **K_A_ d** |  |  |  |  |  | **0.116** | **-0.14** | **0.0784** | **-0.0324** | **0.156** | **-0.116** | **0.238** | **0.0785** |
|  |  |  |  |  |  | **0.145** | **0.0774** | **0.324** | **0.684** | **0.0483** | **0.143** | **0.00254** | **0.323** |
|  |  |  |  |  |  |  |  |  |  |  |  |  |  |
| **K_A_ ax** |  |  |  |  |  |  |  | **-0.0432** | **0.275** | **-0.216** | **-0.00966** | **-0.105** | **0.0798** |
|  |  |  |  |  |  |  |  | **0.588** | **0.000455** | **0.00622** | **0.903** | **0.186** | **0.315** |
|  |  |  |  |  |  |  |  |  |  |  |  |  |  |
| **Na d** |  |  |  |  |  |  |  |  | **-0.0379** | **-0.116** | **0.399** | **0.0869** | **0.192** |
|  |  |  |  |  |  |  |  |  | **0.634** | **0.143** | **0.00000022** | **0.274** | **0.0153** |
|  |  |  |  |  |  |  |  |  |  |  |  |  |  |
| **K_DR_ ax** |  |  |  |  |  |  |  |  |  | **-0.283** | **-0.142** | **-0.0305** | **-0.0194** |
|  |  |  |  |  |  |  |  |  |  | **0.000303** | **0.0727** | **0.701** | **0.807** |
|  |  |  |  |  |  |  |  |  |  |  |  |  |  |
| **g_pas d** |  |  |  |  |  |  |  |  |  |  |  | **0.0318** | **0.322** |
|  |  |  |  |  |  |  |  |  |  |  |  | **0.689** | **0.0000351** |
