# Supplementary material for: The physiological variability of channel density in hippocampal CA1 pyramidal cells and interneurons explored using a unified data-driven modeling workflow
Source: PLoS Comput Biol. 2018 Sep 17;14(9):e1006423. doi: 10.1371/journal.pcbi.1006423 (PMC6160220; doi:10.1371/journal.pcbi.1006423)
Supplement: S7 Table — Only conductances with at least one significant correlation coefficient >|0.25| (gray cells) are shown. The p value corresponding to each coefficient is indicated in italics. (DOCX) [file pcbi.1006423.s008.docx]

|  | **g_pas ax** | **Cagk** | **CaN** | **CaT** | **K_M_ s** | **h** | **Ra d** | **K_M_ ax** | **K_DR_ s** | **K_A_ d** |
| --- | --- | --- | --- | --- | --- | --- | --- | --- | --- | --- |
| **K_DR_ d** | **0.331** | **0.43** | **-0.159** | **-0.221** | **-0.141** | **0.465** | **-0.225** | **0.0977** | **0.142** | **-0.117** |
|  | **0.00528** | **0.000228** | **0.188** | **0.0658** | **0.243** | **0.0000578** | **0.0613** | **0.42** | **0.239** | **0.334** |
| **g_pas ax** |  | **0.638** | **-0.33** | **-0.274** | **-0.0302** | **0.623** | **-0.609** | **0.188** | **0.419** | **-0.55** |
|  |  | **0.0000002** | **0.00542** | **0.0221** | **0.803** | **0.0000002** | **1.35E-08** | **0.119** | **0.000342** | **0.000000997** |
| **Cagk** |  |  | **-0.439** | **-0.402** | **-0.238** | **0.738** | **-0.622** | **0.148** | **0.446** | **-0.331** |
|  |  |  | **0.000163** | **0.000602** | **0.0472** | **0.0000002** | **0.0000002** | **0.219** | **0.000125** | **0.00528** |
| **CaN** |  |  |  | **0.258** | **-0.072** | **-0.209** | **0.233** | **-0.183** | **-0.469** | **0.104** |
|  |  |  |  | **0.031** | **0.553** | **0.0821** | **0.0518** | **0.129** | **0.0000481** | **0.392** |
| **CaT** |  |  |  |  | **0.0117** | **-0.187** | **0.284** | **-0.115** | **-0.112** | **0.054** |
|  |  |  |  |  | **0.923** | **0.121** | **0.0175** | **0.342** | **0.354** | **0.656** |
| **K_M_ s** |  |  |  |  |  | **-0.112** | **-0.071** | **0.405** | **0.0153** | **-0.00353** |
|  |  |  |  |  |  | **0.357** | **0.558** | **0.00055** | **0.9** | **0.977** |
| **h** |  |  |  |  |  |  | **-0.687** | **0.248** | **0.415** | **-0.316** |
|  |  |  |  |  |  |  | **0.0000002** | **0.0384** | **0.000392** | **0.00778** |
| **Ra d** |  |  |  |  |  |  |  | **-0.25** | **-0.34** | **0.312** |
|  |  |  |  |  |  |  |  | **0.0374** | **0.00417** | **0.00869** |

|  | **Na d** | **Na s** | **K_A_ ax** | **K_Ca_** | **CaL** | **K_A_ s** | **g_pas ax** | **K_DR_ ax** | **K_D_** | **Na ax** | **Ra ax** | **e_pas d** | **e_pas ax** |
| --- | --- | --- | --- | --- | --- | --- | --- | --- | --- | --- | --- | --- | --- |
| **K_DR_ d** | **-0.41** | **-0.371** | **-0.0825** | **0.19** | **-0.102** | **0.301** | **-0.0621** | **0.0172** | **0.361** | **0.0933** | **0.24** | **-0.406** | **0.0275** |
|  | **0.000463** | **0.00164** | **0.496** | **0.115** | **0.399** | **0.0115** | **0.608** | **0.887** | **0.00226** | **0.441** | **0.0452** | **0.000533** | **0.821** |
| **g_pas ax** | **-0.0681** | **-0.397** | **0.157** | **0.207** | **-0.0365** | **-0.0422** | **-0.193** | **0.0524** | **0.327** | **0.303** | **0.116** | **-0.421** | **0.059** |
|  | **0.574** | **0.000719** | **0.194** | **0.0854** | **0.763** | **0.728** | **0.11** | **0.666** | **0.00581** | **0.0111** | **0.337** | **0.00031** | **0.627** |
| **Cagk** | **-0.116** | **-0.339** | **0.0412** | **0.278** | **-0.03** | **0.125** | **-0.193** | **0.121** | **0.386** | **0.367** | **0.116** | **-0.486** | **0.0273** |
|  | **0.336** | **0.00427** | **0.734** | **0.0198** | **0.804** | **0.303** | **0.109** | **0.319** | **0.00101** | **0.00187** | **0.338** | **0.0000238** | **0.822** |
| **CaN** | **-0.0209** | **0.16** | **-0.0217** | **-0.111** | **0.0721** | **0.0492** | **0.0345** | **-0.0232** | **-0.202** | **-0.173** | **-0.089** | **-0.0344** | **0.308** |
|  | **0.863** | **0.185** | **0.858** | **0.361** | **0.552** | **0.685** | **0.776** | **0.848** | **0.0941** | **0.153** | **0.463** | **0.777** | **0.00957** |
| **CaT** | **0.0249** | **0.182** | **0.08** | **-0.279** | **0.0599** | **-0.197** | **0.107** | **0.0886** | **-0.087** | **-0.169** | **-0.0829** | **0.181** | **0.0931** |
|  | **0.838** | **0.131** | **0.509** | **0.0194** | **0.621** | **0.101** | **0.375** | **0.465** | **0.473** | **0.161** | **0.494** | **0.133** | **0.442** |
| **K_M_ s** | **0.148** | **-0.062** | **0.0122** | **-0.129** | **0.0969** | **-0.23** | **-0.0766** | **-0.0835** | **-0.076** | **-0.262** | **0.216** | **0.111** | **-0.0052** |
|  | **0.22** | **0.609** | **0.92** | **0.287** | **0.424** | **0.0556** | **0.527** | **0.491** | **0.531** | **0.0283** | **0.0726** | **0.36** | **0.966** |
| **h** | **-0.0631** | **-0.545** | **0.189** | **0.26** | **-0.0925** | **0.0892** | **-0.289** | **0.181** | **0.472** | **0.209** | **0.128** | **-0.609** | **0.112** |
|  | **0.603** | **0.00000137** | **0.117** | **0.0299** | **0.445** | **0.461** | **0.0155** | **0.133** | **0.0000434** | **0.0829** | **0.29** | **1.37E-08** | **0.354** |
| **Ra d** | **-0.0433** | **0.425** | **-0.0462** | **-0.182** | **0.0465** | **-0.0343** | **0.162** | **-0.119** | **-0.337** | **-0.384** | **-0.198** | **0.543** | **-0.0186** |
|  | **0.721** | **0.000272** | **0.703** | **0.132** | **0.701** | **0.777** | **0.181** | **0.327** | **0.00447** | **0.00109** | **0.0997** | **0.00000148** | **0.878** |
| **K_M_ ax** | **0.306** | **-0.23** | **0.0306** | **-0.0777** | **-0.176** | **-0.284** | **-0.343** | **-0.0131** | **0.158** | **-0.106** | **0.176** | **-0.241** | **0.0795** |
|  | **0.0103** | **0.0553** | **0.801** | **0.522** | **0.144** | **0.0173** | **0.00379** | **0.914** | **0.192** | **0.382** | **0.146** | **0.0446** | **0.512** |
| **K_DR_ s** | **-0.232** | **-0.328** | **0.176** | **0.00466** | **-0.0375** | **-0.0558** | **-0.0656** | **0.0137** | **0.198** | **0.301** | **-0.0293** | **-0.301** | **0.104** |
|  | **0.0538** | **0.00573** | **0.145** | **0.969** | **0.757** | **0.646** | **0.589** | **0.91** | **0.1** | **0.0115** | **0.809** | **0.0115** | **0.391** |
| **K_A_ d** | **0.157** | **0.00446** | **-0.0551** | **-0.0671** | **-0.0787** | **-0.0327** | **0.0832** | **-0.0848** | **-0.0577** | **0.188** | **-0.0548** | **0.26** | **0.12** |
|  | **0.195** | **0.971** | **0.649** | **0.58** | **0.516** | **0.787** | **0.492** | **0.484** | **0.634** | **0.119** | **0.651** | **0.0298** | **0.323** |
| **Na s** |  |  | **-0.263** | **-0.295** | **0.208** | **0.139** | **0.392** | **-0.101** | **-0.483** | **-0.0814** | **-0.0572** | **0.439** | **-0.0853** |
|  |  |  | **0.0281** | **0.0135** | **0.0843** | **0.252** | **0.000853** | **0.404** | **0.0000274** | **0.502** | **0.637** | **0.000163** | **0.482** |
| **K_Ca_** |  |  |  |  | **-0.128** | **0.19** | **-0.36** | **-0.0976** | **0.342** | **0.132** | **0.0131** | **-0.145** | **-0.142** |
|  |  |  |  |  | **0.289** | **0.116** | **0.00229** | **0.42** | **0.00393** | **0.277** | **0.914** | **0.232** | **0.241** |
| **CaL** |  |  |  |  |  | **-0.0701** | **0.137** | **-0.264** | **-0.317** | **-0.00895** | **0.131** | **0.155** | **0.0314** |
|  |  |  |  |  |  | **0.563** | **0.259** | **0.0272** | **0.00775** | **0.941** | **0.279** | **0.199** | **0.796** |
| **g_pas ax** |  |  |  |  |  |  |  | **0.0143** | **-0.363** | **0.123** | **-0.0107** | **0.062** | **-0.161** |
|  |  |  |  |  |  |  |  | **0.906** | **0.0021** | **0.31** | **0.929** | **0.609** | **0.182** |
| **K_D_** |  |  |  |  |  |  |  |  |  | **0.0675** | **0.331** | **-0.195** | **-0.0687** |
|  |  |  |  |  |  |  |  |  |  | **0.578** | **0.00534** | **0.106** | **0.571** |
| **Na ax** |  |  |  |  |  |  |  |  |  |  | **0.0972** | **-0.305** | **-0.0119** |
|  |  |  |  |  |  |  |  |  |  |  | **0.423** | **0.0105** | **0.922** |
