# Supplementary material for: The physiological variability of channel density in hippocampal CA1 pyramidal cells and interneurons explored using a unified data-driven modeling workflow
Source: PLoS Comput Biol. 2018 Sep 17;14(9):e1006423. doi: 10.1371/journal.pcbi.1006423 (PMC6160220; doi:10.1371/journal.pcbi.1006423)
Supplement: S8 Table — Only conductances with at least one significant correlation coefficient >|0.25| (gray cells) are shown. The p value corresponding to each coefficient is indicated in italics. (DOCX) [file pcbi.1006423.s009.docx]

|  | **g_pas d** | **h** | **K_M_ ax** | **K_M_ s** | **K_A_ d** | **CaT** | **Na d** | **CaN** | **Na s** | **K_DR_ ax** | **Ra d** |
| --- | --- | --- | --- | --- | --- | --- | --- | --- | --- | --- | --- |
| **K_DR_ d** | **0.317** | **0.338** | **0.247** | **0.179** | **-0.0579** | **-0.0192** | **0.0531** | **0.36** | **-0.315** | **0.212** | **-0.303** |
|  | **0.0137** | **0.0085** | **0.057** | **0.17** | **0.659** | **0.884** | **0.686** | **0.00492** | **0.0145** | **0.103** | **0.0189** |
| **K_DR_ s** | **0.25** | **0.0606** | **0.223** | **0.284** | **-0.141** | **0.0845** | **-0.0337** | **0.221** | **-0.271** | **0.0975** | **-0.198** |
|  | **0.0545** | **0.644** | **0.0864** | **0.0283** | **0.281** | **0.52** | **0.797** | **0.0895** | **0.0366** | **0.457** | **0.129** |
| **g_pas d** |  | **0.292** | **0.254** | **0.317** | **-0.295** | **-0.0192** | **0.227** | **0.127** | **-0.216** | **-0.0104** | **-0.375** |
|  |  | **0.024** | **0.0499** | **0.0138** | **0.0224** | **0.884** | **0.081** | **0.334** | **0.0977** | **0.937** | **0.0033** |
| **h** |  |  | **0.451** | **0.392** | **-0.37** | **-0.0176** | **0.42** | **0.172** | **-0.48** | **0.122** | **-0.183** |
|  |  |  | **0.000328** | **0.00207** | **0.00379** | **0.893** | **0.000906** | **0.188** | **0.00012** | **0.354** | **0.16** |
| **Cagk** |  |  | **-0.001** | **-0.269** | **0.185** | **0.00142** | **0.129** | **-0.057** | **0.17** | **0.000695** | **-0.175** |
|  |  |  | **0.994** | **0.0379** | **0.157** | **0.991** | **0.325** | **0.664** | **0.194** | **0.996** | **0.181** |
| **K_M_ ax** |  |  |  | **0.287** | **-0.337** | **0.315** | **0.194** | **0.354** | **-0.293** | **0.277** | **-0.471** |
|  |  |  |  | **0.0267** | **0.00858** | **0.0144** | **0.137** | **0.00573** | **0.0233** | **0.0322** | **0.000168** |
| **K_M_ s** |  |  |  |  | **-0.614** | **0.0215** | **0.465** | **0.202** | **-0.324** | **0.00995** | **-0.406** |
|  |  |  |  |  | **0.000000165** | **0.87** | **0.000205** | **0.122** | **0.0117** | **0.94** | **0.00136** |
| **K_A_ d** |  |  |  |  |  | **-0.0412** | **-0.407** | **-0.17** | **0.315** | **0.181** | **0.205** |
|  |  |  |  |  |  | **0.754** | **0.00132** | **0.193** | **0.0145** | **0.165** | **0.115** |
| **CaT** |  |  |  |  |  |  | **0.0122** | **0.11** | **0.0574** | **0.0445** | **-0.335** |
|  |  |  |  |  |  |  | **0.926** | **0.402** | **0.662** | **0.735** | **0.00905** |
| **Na d** |  |  |  |  |  |  |  | **-0.00417** | **-0.355** | **0.0635** | **-0.0956** |
|  |  |  |  |  |  |  |  | **0.975** | **0.0056** | **0.629** | **0.466** |
| **CaN** |  |  |  |  |  |  |  |  | **-0.289** | **0.153** | **-0.372** |
|  |  |  |  |  |  |  |  |  | **0.0255** | **0.243** | **0.00358** |
| **Na s** |  |  |  |  |  |  |  |  |  | **-0.323** | **0.12** |
|  |  |  |  |  |  |  |  |  |  | **0.012** | **0.361** |

|  | **g_pas ax** | **K_A_ ax** | **K_A_ s** | **Na ax** | **Ra ax** | **K_D_** | **K_Ca_** | **e_pas d** | **e_pas ax** |
| --- | --- | --- | --- | --- | --- | --- | --- | --- | --- |
| **K_DR_ d** | **-0.314** | **-0.0749** | **-0.0432** | **0.0528** | **-0.138** | **0.228** | **-0.00545** | **-0.323** | **-0.0704** |
|  | **0.0149** | **0.568** | **0.742** | **0.688** | **0.293** | **0.0801** | **0.967** | **0.012** | **0.592** |
| **K_DR_ s** | **0.236** | **0.28** | **-0.172** | **0.127** | **-0.0525** | **0.121** | **0.00283** | **-0.122** | **0.255** |
|  | **0.0691** | **0.0304** | **0.188** | **0.332** | **0.689** | **0.354** | **0.983** | **0.352** | **0.0491** |
| **g_pas d** | **-0.203** | **0.00472** | **-0.149** | **0.287** | **-0.239** | **0.184** | **-0.0374** | **-0.568** | **0.0356** |
|  | **0.12** | **0.971** | **0.256** | **0.0262** | **0.0653** | **0.158** | **0.776** | **0.00000268** | **0.787** |
| **h** | **0.138** | **0.00353** | **-0.224** | **0.117** | **-0.169** | **0.117** | **0.192** | **-0.673** | **-0.0941** |
|  | **0.294** | **0.978** | **0.0845** | **0.371** | **0.197** | **0.373** | **0.141** | **0.0000002** | **0.473** |
| **Cagk** | **-0.000973** | **0.181** | **0.158** | **0.164** | **-0.0673** | **0.291** | **-0.122** | **-0.122** | **0.157** |
|  | **0.994** | **0.166** | **0.226** | **0.211** | **0.608** | **0.0244** | **0.351** | **0.353** | **0.231** |
| **K_M_ ax** | **0.0328** | **-0.0362** | **-0.21** | **0.229** | **-0.095** | **-0.0635** | **0.0925** | **-0.354** | **0.166** |
|  | **0.803** | **0.783** | **0.107** | **0.079** | **0.469** | **0.629** | **0.481** | **0.00574** | **0.205** |
| **K_M_ s** | **-0.00871** | **-0.0898** | **-0.106** | **0.392** | **-0.0545** | **0.0116** | **-0.115** | **-0.394** | **-0.0382** |
|  | **0.947** | **0.493** | **0.418** | **0.00203** | **0.678** | **0.929** | **0.379** | **0.00197** | **0.771** |
| **K_A_ d** | **-0.0109** | **0.203** | **0.169** | **-0.2** | **-0.0904** | **0.33** | **0.0944** | **0.465** | **0.0757** |
|  | **0.934** | **0.119** | **0.196** | **0.125** | **0.491** | **0.0101** | **0.472** | **0.000201** | **0.564** |
| **CaL** | **-0.173** | **0.0283** | **0.302** | **0.0936** | **-0.261** | **0.316** | **0.162** | **-0.125** | **0.0432** |
|  | **0.186** | **0.829** | **0.0191** | **0.475** | **0.0443** | **0.0141** | **0.216** | **0.34** | **0.742** |
| **Na d** | **-0.0369** | **-0.0711** | **-0.109** | **0.272** | **-0.102** | **-0.0763** | **0.0138** | **-0.458** | **0.0154** |
|  | **0.779** | **0.588** | **0.407** | **0.0357** | **0.436** | **0.561** | **0.916** | **0.000264** | **0.907** |
| **CaN** | **-0.142** | **-0.105** | **-0.0458** | **0.127** | **-0.0573** | **0.0209** | **-0.309** | **-0.0551** | **-0.142** |
|  | **0.279** | **0.425** | **0.727** | **0.331** | **0.663** | **0.874** | **0.0165** | **0.675** | **0.276** |
| **Na s** | **-0.116** | **-0.0268** | **0.21** | **-0.0344** | **-0.143** | **0.0503** | **-0.0532** | **0.336** | **0.0997** |
|  | **0.375** | **0.839** | **0.107** | **0.793** | **0.276** | **0.702** | **0.686** | **0.00894** | **0.447** |
| **Ra d** | **0.0674** | **0.0499** | **0.185** | **-0.262** | **-0.00275** | **-0.0775** | **0.268** | **0.295** | **-0.081** |
|  | **0.607** | **0.704** | **0.157** | **0.0435** | **0.983** | **0.555** | **0.0384** | **0.0223** | **0.537** |
| **K_A_ s** |  |  |  | **0.117** | **0.111** | **0.264** | **-0.174** | **0.328** | **-0.102** |
|  |  |  |  | **0.371** | **0.399** | **0.0417** | **0.184** | **0.0108** | **0.437** |
| **Na ax** |  |  |  |  | **-0.0363** | **0.189** | **-0.137** | **-0.163** | **0.285** |
|  |  |  |  |  | **0.782** | **0.147** | **0.295** | **0.213** | **0.0277** |
| **Ra ax** |  |  |  |  |  | **-0.15** | **-0.255** | **0.18** | **0.173** |
|  |  |  |  |  |  | **0.25** | **0.0496** | **0.169** | **0.185** |
