# Supplementary material for: The physiological variability of channel density in hippocampal CA1 pyramidal cells and interneurons explored using a unified data-driven modeling workflow
Source: PLoS Comput Biol. 2018 Sep 17;14(9):e1006423. doi: 10.1371/journal.pcbi.1006423 (PMC6160220; doi:10.1371/journal.pcbi.1006423)
Supplement: S9 Table — Only conductances with at least one significant correlation coefficient >|0.25| (gray cells) are shown. The p value corresponding to each coefficient is indicated in italics. (DOCX) [file pcbi.1006423.s010.docx]

|  | **g_pas ax** | **Cagk** | **CaN** | **CaT** | **K_M_ s** | **h** | **Ra d** | **K_M_ ax** | **K_DR_ s** | **K_A_ d** | **Na d** | **Na s** |
| --- | --- | --- | --- | --- | --- | --- | --- | --- | --- | --- | --- | --- |
| **K_DR_ d** | **0.331** | **0.43** | **-0.159** | **-0.221** | **-0.141** | **0.465** | **-0.225** | **0.0977** | **0.142** | **-0.117** | **-0.41** | **-0.371** |
|  | ***0.00528*** | ***0.000228*** | ***0.188*** | ***0.0658*** | ***0.243*** | ***0.0000578*** | ***0.0613*** | ***0.42*** | ***0.239*** | ***0.334*** | ***0.000463*** | ***0.00164*** |
| **g_pas ax** |  | **0.638** | **-0.33** | **-0.274** | **-0.0302** | **0.623** | **-0.609** | **0.188** | **0.419** | **-0.55** | **-0.0681** | **-0.397** |
|  |  | ***0.0000002*** | ***0.00542*** | ***0.0221*** | ***0.803*** | ***0.0000002*** | ***1.35E-08*** | ***0.119*** | ***0.000342*** | ***9.97E-07*** | ***0.574*** | ***0.000719*** |
| **Cagk** |  |  | **-0.439** | **-0.402** | **-0.238** | **0.738** | **-0.622** | **0.148** | **0.446** | **-0.331** | **-0.116** | **-0.339** |
|  |  |  | ***0.00016*** | ***0.000602*** | ***0.0472*** | ***0.0000002*** | ***0.0000002*** | ***0.219*** | ***0.000125*** | ***0.00528*** | ***0.336*** | ***0.00427*** |
| **CaN** |  |  |  | **0.258** | **-0.072** | **-0.209** | **0.233** | **-0.183** | **-0.469** | **0.104** | **-0.0209** | **0.16** |
|  |  |  |  | ***0.031*** | ***0.553*** | ***0.0821*** | ***0.0518*** | ***0.129*** | ***4.81E-05*** | ***0.392*** | ***0.863*** | ***0.185*** |
| **CaT** |  |  |  |  | **0.0117** | **-0.187** | **0.284** | **-0.115** | **-0.112** | **0.054** | **0.0249** | **0.182** |
|  |  |  |  |  | ***0.923*** | ***0.121*** | ***0.0175*** | ***0.342*** | ***0.354*** | ***0.656*** | ***0.838*** | ***0.131*** |
| **K_M_ s** |  |  |  |  |  | **-0.112** | **-0.071** | **0.405** | **0.0153** | **-0.00353** | **0.148** | **-0.062** |
|  |  |  |  |  |  | ***0.357*** | ***0.558*** | ***0.00055*** | ***0.9*** | ***0.977*** | ***0.22*** | ***0.609*** |
| **h** |  |  |  |  |  |  | **-0.687** | **0.248** | **0.415** | **-0.316** | **-0.0631** | **-0.545** |
|  |  |  |  |  |  |  | ***0.0000002*** | ***0.0384*** | ***0.000392*** | ***0.00778*** | ***0.603*** | ***1.37E-06*** |
| **Ra d** |  |  |  |  |  |  |  | **-0.25** | **-0.34** | **0.312** | **-0.0433** | **0.425** |
|  |  |  |  |  |  |  |  | ***0.0374*** | ***0.00417*** | ***0.00869*** | ***0.721*** | ***0.000272*** |
| **K_M_ ax** |  |  |  |  |  |  |  |  | **0.0845** | **-0.0464** | **0.306** | **-0.23** |
|  |  |  |  |  |  |  |  |  | ***0.486*** | ***0.702*** | ***0.0103*** | ***0.0553*** |
| **K_DR_ s** |  |  |  |  |  |  |  |  |  | **-0.106** | **-0.232** | **-0.328** |
|  |  |  |  |  |  |  |  |  |  | ***0.382*** | ***0.0538*** | ***0.00573*** |

|  | **K_A_ ax** | **K_Ca_ d** | **K_A_ s** | **g_pas ax** | **K_DR_ ax** | **K_D_** | **Na ax** | **Ra ax** | **e_pas d** | **e_pas ax** |
| --- | --- | --- | --- | --- | --- | --- | --- | --- | --- | --- |
| **K_DR_ d** | **-0.0825** | **0.19** | **0.301** | **-0.0621** | **0.0172** | **0.361** | **0.0933** | **0.24** | **-0.406** | **0.0275** |
|  | ***0.496*** | ***0.115*** | ***0.0115*** | ***0.608*** | ***0.887*** | ***0.00226*** | ***0.441*** | ***0.0452*** | ***0.000533*** | ***0.821*** |
| **g_pas ax** | **0.157** | **0.207** | **-0.0422** | **-0.193** | **0.0524** | **0.327** | **0.303** | **0.116** | **-0.421** | **0.059** |
|  | ***0.194*** | ***0.0854*** | ***0.728*** | ***0.11*** | ***0.666*** | ***0.00581*** | ***0.0111*** | ***0.337*** | ***0.00031*** | ***0.627*** |
| **Cagk** | **0.0412** | **0.278** | **0.125** | **-0.193** | **0.121** | **0.386** | **0.367** | **0.116** | **-0.486** | **0.0273** |
|  | ***0.734*** | ***0.0198*** | ***0.303*** | ***0.109*** | ***0.319*** | ***0.00101*** | ***0.00187*** | ***0.338*** | ***0.0000238*** | ***0.822*** |
| **CaN** | **-0.0217** | **-0.111** | **0.0492** | **0.0345** | **-0.0232** | **-0.202** | **-0.173** | **-0.089** | **-0.0344** | **0.308** |
|  | ***0.858*** | ***0.361*** | ***0.685*** | ***0.776*** | ***0.848*** | ***0.0941*** | ***0.153*** | ***0.463*** | ***0.777*** | ***0.00957*** |
| **CaT** | **0.08** | **-0.279** | **-0.197** | **0.107** | **0.0886** | **-0.087** | **-0.169** | **-0.0829** | **0.181** | **0.0931** |
|  | ***0.509*** | ***0.0194*** | ***0.101*** | ***0.375*** | ***0.465*** | ***0.473*** | ***0.161*** | ***0.494*** | ***0.133*** | ***0.442*** |
| **K_M_ s** | **0.0122** | **-0.129** | **-0.23** | **-0.0766** | **-0.0835** | **-0.076** | **-0.262** | **0.216** | **0.111** | **-0.0052** |
|  | ***0.92*** | ***0.287*** | ***0.0556*** | ***0.527*** | ***0.491*** | ***0.531*** | ***0.0283*** | ***0.0726*** | ***0.36*** | ***0.966*** |
| **h** | **0.189** | **0.26** | **0.0892** | **-0.289** | **0.181** | **0.472** | **0.209** | **0.128** | **-0.609** | **0.112** |
|  | ***0.117*** | ***0.0299*** | ***0.461*** | ***0.0155*** | ***0.133*** | ***0.0000434*** | ***0.0829*** | ***0.29*** | ***1.37E-08*** | ***0.354*** |
| **Ra d** | **-0.0462** | **-0.182** | **-0.0343** | **0.162** | **-0.119** | **-0.337** | **-0.384** | **-0.198** | **0.543** | **-0.0186** |
|  | ***0.703*** | ***0.132*** | ***0.777*** | ***0.181*** | ***0.327*** | ***0.00447*** | ***0.00109*** | ***0.0997*** | ***0.00000148*** | ***0.878*** |
| **K_M_ ax** | **0.0306** | **-0.0777** | **-0.284** | **-0.343** | **-0.0131** | **0.158** | **-0.106** | **0.176** | **-0.241** | **0.0795** |
|  | ***0.801*** | ***0.522*** | ***0.0173*** | ***0.00379*** | ***0.914*** | ***0.192*** | ***0.382*** | ***0.146*** | ***0.0446*** | ***0.512*** |
| **K_DR_ s** | **0.176** | **0.00466** | **-0.0558** | **-0.0656** | **0.0137** | **0.198** | **0.301** | **-0.0293** | **-0.301** | **0.104** |
|  | ***0.145*** | ***0.969*** | ***0.646*** | ***0.589*** | ***0.91*** | ***0.1*** | ***0.0115*** | ***0.809*** | ***0.0115*** | ***0.391*** |
| **K_A_ d** | **-0.0551** | **-0.0671** | **-0.0327** | **0.0832** | **-0.0848** | **-0.0577** | **0.188** | **-0.0548** | **0.26** | **0.12** |
|  | ***0.649*** | ***0.58*** | ***0.787*** | ***0.492*** | ***0.484*** | ***0.634*** | ***0.119*** | ***0.651*** | ***0.0298*** | ***0.323*** |
| **Na s** | **-0.263** | **-0.295** | **0.139** | **0.392** | **-0.101** | **-0.483** | **-0.0814** | **-0.0572** | **0.439** | **-0.0853** |
|  | ***0.0281*** | ***0.0135*** | ***0.252*** | ***0.000853*** | ***0.404*** | ***0.0000274*** | ***0.502*** | ***0.637*** | ***0.000163*** | ***0.482*** |
| **K_Ca_** |  |  | **0.19** | **-0.36** | **-0.0976** | **0.342** | **0.132** | **0.0131** | **-0.145** | **-0.142** |
|  |  |  | ***0.116*** | ***0.00229*** | ***0.42*** | ***0.00393*** | ***0.277*** | ***0.914*** | ***0.232*** | ***0.241*** |
| **CaL** |  |  | **-0.0701** | **0.137** | **-0.264** | **-0.317** | **-0.00895** | **0.131** | **0.155** | **0.0314** |
|  |  |  | ***0.563*** | ***0.259*** | ***0.0272*** | ***0.00775*** | ***0.941*** | ***0.279*** | ***0.199*** | ***0.796*** |
| **g_pas ax** |  |  |  |  | **0.0143** | **-0.363** | **0.123** | **-0.0107** | **0.062** | **-0.161** |
|  |  |  |  |  | ***0.906*** | ***0.0021*** | ***0.31*** | ***0.929*** | ***0.609*** | ***0.182*** |
| **K_D_** |  |  |  |  |  |  | **0.0675** | **0.331** | **-0.195** | **-0.0687** |
|  |  |  |  |  |  |  | ***0.578*** | ***0.00534*** | ***0.106*** | ***0.571*** |
| **Na ax** |  |  |  |  |  |  |  | **0.0972** | **-0.305** | **-0.0119** |
|  |  |  |  |  |  |  |  | ***0.423*** | ***0.0105*** | ***0.922*** |
